# Genome‐Wide Association Studies of Delay Discounting and Impulsive Personality Traits in Children From the Adolescent Behavior and Cognitive Development Study

**DOI:** 10.1111/gbb.70033

**Published:** 2025-08-23

**Authors:** Wei Q. Deng, Mahmoud Elsayed, Kyla L. Belisario, Sandra Sanchez‐Roige, Abraham A. Palmer, James MacKillop

**Affiliations:** ^1^ Peter Boris Centre for Addictions Research, St. Joseph's Healthcare Hamilton Hamilton Ontario Canada; ^2^ Department of Psychiatry and Behavioural Neurosciences McMaster University Hamilton Ontario Canada; ^3^ Department of Psychiatry UCSD La Jolla California USA; ^4^ Division of Genetic Medicine Vanderbilt University Medical Center Nashville Tennessee USA; ^5^ Institute for Genomic Medicine, UCSD La Jolla California USA

## Abstract

Impulsivity, often operationalized as delay discounting (DD) and as impulsive personality traits via the UPPS‐P scales, is a key transdiagnostic construct across psychiatric disorders. Recent genome‐wide association studies (GWAS) have studied the genetic basis of impulsivity in adults, but it remains unclear how similar the genetic architecture of DD is in children. The present study conducted GWAS of DD and impulsivity traits in 5548 children (ages 9–10 years old) of genetically inferred European ancestry from the Adolescent Brain Cognitive Development (ABCD) Study. Heritability estimates for DD (*h* ^2^ = 0.20, S.E. = 0.10) and UPPS‐P subscales (*h* ^2^ = 0.08–0.11 S.E. = 0.05) were comparable to adult estimates. Genetic correlations between adult and child impulsivity were modest (*r*_g_ = 0.28–0.46), with positive urgency showing the strongest correlation (*r*_g_ = 0.83). While no genome‐wide significant associations were identified, the top associated variants were mapped to genes previously linked to smoking initiation (rs3820908; *p* = 6.5 × 10^−8^) and UPPS‐P Lack of Premeditation (rs17292179; *p* = 4.2 × 10^−7^). Polygenic score (PGS) associations were used to compare the genetic signals in children with those reported in adults. Adult PGSs for DD and positive and negative urgency indicators explained small but significant variance in the respective child impulsivity phenotypes (0.36%–0.44%, *p* < 7.5 × 10^−4^). Additionally, UPPS‐P indices were broadly associated with PGSs derived from adult externalizing (0.42%–1.02%) and ADHD (0.23%–0.79%). This first GWAS of impulsivity in children offers a developmentally informed comparison of genetic influences, revealing both similarities and differences by developmental stage.

## Graphical abstract

This is the first GWAS of impulsivity in children and the first to examine impulsivity genetics from a life‐course perspective, revealing partial genetic overlap between children and adult traits. Leveraging polygenic overlap and shared liability across behavioral traits may enhance the prediction of impulsivity in children beyond trait‐specific genetic signals.

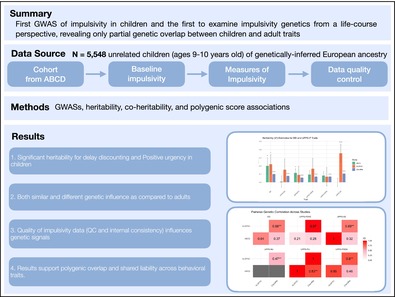

## 1 Introduction

Impulsivity, broadly defined as lack of self‐control, is associated with a host of adverse health outcomes [1, 2, 3] and may function as intermediate mechanisms, or endophenotype, through which genetic variation confers risk [4, 5, 6]. However, impulsivity is not a unitary trait [7, 8] and several specific measures of impulsivity are particularly promising endophenotypes for understanding substance use disorders (SUD), behavioral addictions, and other psychiatric disorders [4, 9, 10]. Of a number of options, some of the most studied are delay discounting (DD), referring to immediate reward preference [11], and the five‐factor UPPS‐P impulsive behavior scales [12], referring to several distinct dispositions. Indeed, steeper DD and higher UPPS‐P impulsivity scores [13] have been shown to precede the onset of SUDs, with prospective studies indicating that impulsive traits in childhood and adolescence predict later substance use initiation, escalation, and addiction risk [3, 14, 15]. Unlike task‐based DD measures, the UPPS‐P scale [13] measures in five self‐reported impulsive personality subdomains—Negative Urgency (e.g., propensity to act rashly during negative mood states), Positive Urgency (e.g., acting out during positive mood states), Lack of Premeditation (e.g., acting without thinking about the consequences), Lack of Perseverance (e.g., difficulty staying focused or giving up easily when challenged), and Sensation Seeking (e.g., the tendency to seek out new, exciting, or risky experiences)—providing a more nuanced framework for assessing different facets of impulsivity. These facets are distinctively associated with various aspects of substance use, with Sensation Seeking and Negative Urgency particularly implicated in the age of initiation [16, 17] and frequency of use [18]. Twin studies have demonstrated DD [19, 20] and facets of impulsive personality scales [21, 22] are heritable in adolescents (~19%–56%) and adults (20%–64%), making them valuable markers for dissecting genetic studies of SUD and other behavioral problems. These phenotypes also have transdiagnostic significance, showing both positive and negative correlations with several psychiatric disorders [4, 9, 23, 24, 25, 26].

Recent studies on the genetic basis of substance use [27, 28, 29, 30, 31, 32, 33, 34, 35], multiple forms of impulsivity [36, 37, 38, 39, 40], and more broadly self‐regulation [41] have made significant strides, particularly through large‐scale genome‐wide association studies (GWAS) in the adult population. SNP‐based heritability estimates for measures of impulsivity range from 5% to 10%, confirming the additive genetic component that influences these traits. Several genome‐wide significant findings for DD and UPPS‐P revealed a broad influence on other behavioral traits [25, 40, 42], many of which are relevant to SUD [43]. Notably, there was also overlap in the significant genetic signals between measures of impulsivity; for example, genetic variants in the *CADM2* were significantly associated with the Sensation Seeking subscale of the UPPS‐P model, as well as non‐planning and motor from the Barratt Impulsive Scale version 11 [44], and drug experimentation [39].

Advances in the genetics of impulsivity in adults highlight the complexity and importance of these traits as transdiagnostic markers [24, 25]. Building on these findings, there is a pressing need to explore how these genetic components manifest in children, particularly during critical developmental stages. Phenotypic characterization of DD and the UPPS‐P have been studied in children and adolescents [45, 46, 47, 48, 49], establishing that impulsivity is not only present and multi‐faceted but also highly malleable [50, 51, 52]. Persistent and elevated impulsivity are often embedded within a broader externalizing spectrum, which includes behaviors such as rule‐breaking, aggression, substance use, and psychiatric conditions, such as Attention‐Deficit/Hyperactivity Disorder (ADHD). Externalizing traits may share developmental pathways with impulsivity and contribute similarly to a range of psychiatric and behavioral outcomes [53, 54, 55]. Understanding these traits during this critical cognitive developmental period offers valuable insights into the trajectory of impulsivity and its impact on behavior with limited substance exposure. Thus, a genome‐wide investigation of impulsivity in children offers several key benefits and addresses important gaps in our knowledge. First, it can provide insights into the polygenic contribution to impulsivity in childhood and allow comparisons with adults to assess developmental stability or change in genetic influences [56]. Second, similar to other complex traits, GWAS in children may contribute to a better understanding of the genetic architecture through the identified signals. For example, genetic variants may exhibit higher penetrance [57, 58, 59], meaning that the likelihood of expressing the trait in the presence of an associated allele is higher in younger individuals. Finally, given the strong connection between impulsivity and brain development [50, 60], it is essential to understand how genetic variations affect both the tendency for impulsive decision‐making and the structural and functional characteristics of the brain.

The Adolescent Brain Cognitive Development (ABCD) study [61] presents an opportunity to simultaneously examine behavioral, neuropsychological, and psychosocial measures in relation to genetic variants in a cohort of 11,875 youths of diverse backgrounds. Our primary aim was to identify genetic variants associated with rigorously QCed DD and UPPS‐P traits in children. Our secondary aim was to estimate the SNP‐based heritability and genetic correlation between impulsivity indicators in children and contrast them with those derived from external GWASs based on adult populations. Building on these, we sought to validate the associations between impulsivity indicators in children and polygenic scores (PGS) of impulsivity and related phenotypes derived from adult GWASs.

## 2 Material and Methods

### 2.1 Study Population

The Adolescent Brain Cognitive Development (ABCD) study is an ongoing longitudinal study of brain development and behavioral health that is following more than 11,000 children in the United States for up to 10 years [61]. The study recruited children aged between 9 and 10 years old at baseline from sampled schools from 21 sites across the United State [62]. Written informed consent was obtained from parents or guardians. A wide range of data, including demographics, mental and physical health assessments from each follow‐up, multi‐omics, and brain imaging, are available [63, 64, 65]. This investigation utilized data from release 5.0, focusing on the full cohort, the updated genomic data, and phenotypic measurements in the largest subset of 5548 children of genetically inferred European ancestry based on reference populations.

### 2.2 DD Phenotype Assessment

DD was assessed during the first follow‐up visit (1‐year after the baseline visit) using a computer‐based adjusting‐amount delay discounting procedure adapted for children [66, 67]. Participating children were presented with 42 hypothetical monetary choices (7‐item that is administered 6 times), where they had to decide between receiving a smaller immediate reward (of adjusting magnitude) or a larger reward of $100 after different delay intervals (6 h, 1 day, 1 week, 1 month, 3 months, 1 year, and 5 years). Refer to the manual (https://www.millisecond.com/download/library/v6/delaydiscountingtask/) for the approach followed in ABCD. Several studies have raised issues with the quality of the DD phenotype in ABCD [48, 49, 68], highlighting the need for robust quality control (QC) in this sample. A hyperbolic discounting model [11] was used to estimate the discounting rate (i.e., the *k*‐value), and the quality of DD was evaluated using model fit metrics. Specifically, different QC thresholds for the hyperbolic regression model fit R^2^ were investigated, taking into account the trade‐off between data quality and the potential reduction in sample size [69]. Further details on phenotype quality control and data processing can be found in the Supporting Information. The hyperbolic regression model fit *R* ^2^ cut‐off was chosen to be ≥ 0.5, reflecting more than half of the variance explained. As a sensitivity analysis, we repeated the heritability estimation for DD across different hyperbolic regression model fit *R* ^2^ thresholds. In addition to the primary threshold of *R* ^2^ = 0.5, we also examined *R* ^2^ = 0 (no removal), 0.1, 0.2, 0.3, 0.4, 0.6, 0.7, 0.8, and 0.9 to assess whether the heritability estimate of DD was influenced by the choice of model fit threshold. Prior to analysis, DD was log10‐transformed and winsorized by replacing values beyond four standard deviations with the nearest non‐extreme values.

### 2.3 UPPS‐P Phenotype Assessment

The UPPS‐P impulsive personality scale was administered to assess the five subscales of impulsive personality during the baseline collection. A child‐friendly version of the UPPS‐P was used, with 20 items simplified for age‐appropriate understanding [63]. Responses were collected on a 4‐point Likert scale ranging from “Not at all like me” to “Very much like me.” We then summed up the respective items without further quality control to calculate Negative Urgency, Positive Urgency, Sensation Seeking, Lack of Planning, and Lack of Perseverance, respectively.

### 2.4 Genotyping and Quality Control

Genomic DNA was extracted from saliva samples using the SmokeScreen targeted array based on the Affymetrix Axiom platform [70]. For autosomes and the X chromosome, we used the imputed genotypes as part of the most recent data (ABCD Data Releases 5.0; *n* = 11,666), which included stringent QCs. Briefly, samples with mismatched genetic and reported sex were removed (*n* = 185), retaining only unrelated individuals (removing *n* = 3490) that were genetically similar to the European superpopulations (removing *n* = 2443) from the 1000 Genomes Project [71] (Figure S1). We focused on participants in the European genetic similarity group because of the: (1) limited statistical power in non‐European genetic similarity groups, (2) potential bias for trans‐ancestry meta‐analysis with unbalanced sample [72], (3) reduced accuracy of European‐derived polygenic risk scores in other genetic ancestries [73]. While this approach limits generalizability, it ensures methodological rigor and interpretability of genetic associations. We then applied additional QC steps to retain sample and genetic variants with missing rate < 0.05, genetic variants with minor allele frequency (MAF) > 0.05, and for autosomal SNPs only: Hardy–Weinberg disequilibrium *p*‐value > 5 × 10^−7^. After quality control, 5548 unrelated, genetically inferred European samples remained in the analysis. Sample and SNP inclusion and exclusion criteria are summarized in Figure S2.

### 2.5 Heritability and Genetic Correlation

SNP‐based heritability in ABCD was conducted using genome‐based restricted maximum likelihood (GREML) via GCTA [74] on individual‐level data and alternatively via LDSC [75] using summary‐level data. The model adjusted for continuous covariates, including children's age, their genetic sex, and the first 10 genetic principal components (PCs). While GREML is preferable over methods using summary‐level data [76], LDSC has the flexibility to handle genetic correlation analyses using external summary‐level data. Thus, we computed genetic correlations of impulsivity traits using GWAS summary statistics derived in ABCD via LDSC and compared them to reported genetic correlations from two external GWASs. These include 3860 young adults aged 24 to 266 from the Avon Longitudinal Study of Parents and Children (ALSPAC) study [77] and over 135,000 adults from the general population that provided consent to participate in 23andMe research [38, 40](Table S1). For each impulsivity trait, we further examined pairwise genetic correlation across the three studies using LDSC.

### 2.6 Statistical Analyses

Autosomal GWAS was conducted in PLINK2 [78] using a linear regression model to test the association between the genotype of each SNP, coded additively by the number of minor alleles, and the impulsivity phenotype, while accounting for genetic sex, age at the time of data collection, and the first 10 genetic PCs. Association results for SNPs of interest and their nearby variants were visualized using LocusZoom [79, 80]. For Xchr association analyses, we employed a linear regression model using a 3 degree freedom test for Xchr variants [81] that is robust to male genotype coding and X‐inactivation, while adjusting for the same set of covariates as the autosomal model.

Gene‐level associations for autosome and Xchr were conducted using MAGMA v1.10 [82] using variant‐level *p*‐values and the matching European LD reference panel curated from phase 3 of the 1000 Genomes Project [71]. SNPs were mapped to genes based on their genomic location (genome build 38), and the gene‐based *p*‐values were computed using the SNP‐wise mean model.

Polygenic scores (PGS) were derived using the LDpred2‐auto method [83], which infers the Bayesian posterior distributions of effect size and adaptively evaluates hyperparameters without the validation phenotype as input. GWAS summary statistics from prior large‐scale studies were curated for DD [40], UPPS‐P [39], adventurousness [84], ADHD [85], and the externalizing factor [41] and (Table S2). We removed variants that mapped to multiple genomic locations (i.e., INDELs), SNPs that had inconsistent reference and alternative alleles between the target population (i.e., ABCD) and external GWAS summary statistics. Each PGS was standardized to have mean zero and unit variance. The performances of PGSs were assessed using the difference in adjusted *R* ^2^ between regression models with and without the PGS, accounting for age, genetic sex, and the first 10 genetic PCs. We applied a false‐discovery rate (FDR) correction to control for multiple hypothesis testing and considered results with an FDR adjusted *p*‐value < 0.05 as significant. We further examined whether trait‐specific PGSs overlapped with other adult GWAS‐derived PGSs in explaining trait variance by comparing the adjusted *R* ^2^ from the following covariate‐adjusted models to a covariate‐only model: (1) trait PGS; (2) all PGSs; (3) all PGS excluding externalizing; and additionally, (4) both trait and externalizing PGSs versus a trait PGS only covariate‐adjusted model. All statistical analyses were conducted in R version 4.1.0 [86].

## 3 Results

### 3.1 Sample Characteristics

The demographic characteristics of the ABCD genetic subsample by sex are in Table 1. Empirical distribution of DD and UPPS‐P subscales (Figure S3) demonstrated widespread sex differences across the impulsivity phenotypes, with males (*n* = 2959) generally more impulsive than females (*n* = 2589). Phenotypic correlation patterns were similar between ABCD children and other adult populations (Figure S4B), with moderate positive correlations between the two urgency indicators (*r*_range_ = 0.48–0.61) and between Lack of Premeditation and Lack of Perseverance (*r*_range_ = 0.41–0.44). While DD generally exhibited weaker correlations with UPPS‐P facets, the only consistent correlations observed for DD across the three studies (ABCD, ALSPAC, and 23andMe) were with Positive Urgency (*r*_range_ = 0.11–0.19) and Sensation Seeking (*r*_range_ = −0.10 to −0.04). As expected, children in ABCD and young adults in ALSPAC both exhibited higher levels of impulsivity across all traits than the general adult population (Table S1).

**TABLE 1:** Summary of phenotypes and demographic variables stratified by sex subgroups in ABCD children.

	Boys	Girls	Overall	
Delay Discounting logk	(*N* = 2959)	Girls (*N* = 2589)	(*N* = 5548)	Two‐sample *p*‐value
Age at Baseline				
Mean (SD)	10.9 (0.764)	10.9 (0.759)	10.9 (0.762)	0.25
Parental income				
< $5 k	26 (0.9%)	21 (0.8%)	47 (0.8%)	0.67
$5 k‐11,999	46 (1.6%)	27 (1.0%)	73 (1.3%)	
$12 k‐15,999	27 (0.9%)	23 (0.9%)	50 (0.9%)	
$16 k‐24,999	67 (2.3%)	68 (2.6%)	135 (2.4%)	
$25 k‐34,999	93 (3.1%)	88 (3.4%)	181 (3.3%)	
$35 k‐49,999	172 (5.8%)	157 (6.1%)	329 (5.9%)	
$50 k‐74,999	388 (13.1%)	349 (13.5%)	737 (13.3%)	
$75 k‐99,999	461 (15.6%)	429 (16.6%)	890 (16.0%)	
$100 k‐199,999	1111 (37.5%)	935 (36.1%)	2046 (36.9%)	
$200 k+	390 (13.2%)	369 (14.3%)	759 (13.7%)	
Mean (SD)	−2.09 (0.702)	−2.20 (0.704)	−2.14 (0.71)	< 0.001
Negative Urgency				
Mean (SD)	8.61 (2.59)	8.15 (2.56)	8.40 (2.59)	< 0.001
Positive Urgency				
Mean (SD)	7.97 (2.82)	7.47 (2.80)	7.74 (2.82)	< 0.001
Sensation Seeking				
Mean (SD)	10.3 (2.61)	9.61 (2.61)	9.96 (2.63)	< 0.001
Lack of Premeditation				
Mean (SD)	8.13 (2.34)	7.57 (2.27)	7.87 (2.33)	< 0.001
Lack of Perseverance				
Mean (SD)	7.18 (2.18)	6.96 (2.23)	7.08 (2.21)	< 0.001

### 3.2 SNP‐Based Heritability and Genetic Correlations

**FIGURE 1:**
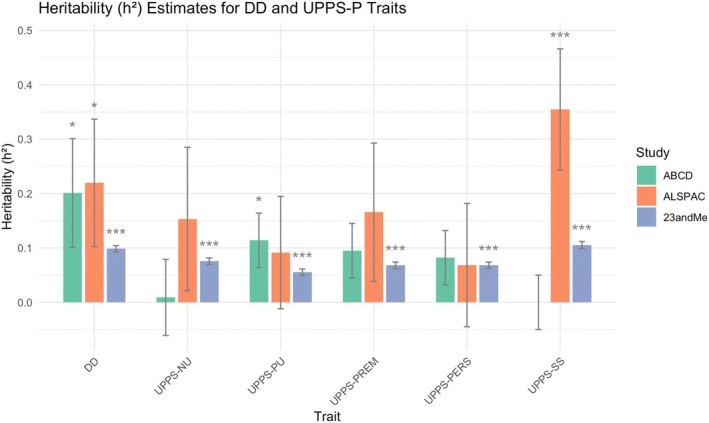
Estimated SNP‐based heritability for impulsivity across three population studies. The bar plot summarizes the SNP‐based heritability (*h* ^2^) estimates for delay discounting and the five UPPS‐P impulsivity subscales across three cohorts: ABCD, ALSPAC, and 23andMe. Heritability estimates for ABCD was based on GCTA results, and those for ALSPAC and 23andMe were derived using LDSC. Asterisks indicate statistically significant estimates (*p* < 0.05, *p* < 0.01, *p* < 0.001), with several traits showing significant heritability across multiple cohorts, particularly delay discounting.

The estimated SNP‐based heritability via GREML‐GCTA was statistically significant for DD (*h* ^2^ = 0.20, standard error = 0.10, *p* = 0.04) and Positive Urgency (*h* ^2^ = 0.11, S.E. = 0.05, *p* = 0.04; Figure 1). Except for Negative Urgency and Sensation Seeking, which did not show significant heritability, the remaining UPPS‐P indicators had moderate heritability estimates between 0.082 and 0.0955 (S.E. = 0.05; Figure 1; Table 2). The LDSC estimates were similar to GREML but with much larger standard errors (Table S3) and did not yield any significant results. Across the three studies, we found estimated heritability of DD to be most robustly supported (Figure 1). There was also moderate consistency in Positive Urgency, with significant heritability estimates in both ABCD and 23andMe samples, and in Sensation Seeking, with significant estimates in ALSPAC and 23andMe (Figure 1). For DD, the estimated heritability was not sensitive to different model fit thresholds, with estimates ranging between 0.12 and 0.22 (GCTA) and increasing S.E. as the number of samples being removed at more stringent thresholds (Table S3).

**TABLE 2:** Estimated SNP‐based heritability using GCTA.

Impulsivity trait	Estimate *h* ^2^	S.E.	*p*‐value (two‐sided)
Delay Discounting	0.201	0.10	0.038
Negative Urgency	0.009	0.07	0.902
Positive Urgency	0.114	0.05	0.038
Sensation Seeking	0.000	0.05	1.000
Lack of Perseverance	0.082	0.05	0.128
Lack of Premeditation	0.095	0.05	0.056

There was one marginally significant LDSC genetic correlation between Lack of Premeditation and Lack of Perseverance (*r*_g_ = 0.97, S.E. = 0.46, *p* = 0.04; Table S4) in ABCD. This was also the only consistent finding across studies, similar to the *r*_g_ = 1 reported in ALSPAC and *r*_g_ = 0.5 reported in 23andMe (Figure S4). Specifically, when examining the genetic correlation of the same impulsivity trait across studies, there was stronger agreement between 23andMe and young adults in ALSPAC, with 4 out of 6 measures showing significant correlations (*r*_g_ = 0.47–0.80; Figure S5). In contrast, the genetic correlation between 23andMe and ABCD was significant only for Positive Urgency (*r*_g_ = 0.83, S.E. = 0.34; Table S5).

### 3.3 Genome‐Wide Association Studies in ABCD

As expected given the modest sample size, the GWAS did not yield any genome‐wide significant markers (Tables S6 and S7; Figure S6), with no sign of type I error inflation (Figure S7) and the genomic control lambdas were within an acceptable range (1.001–1.019). There was one signal just below the genome‐wide significance: between DD and variants in the *HECW2* gene on chromosome 2 (lead SNP rs3820908; *p* = 6.5 × 10^−8^; MAF = 0.29), and three suggestive signals with *p* < 1 × 10^−7^: between Negative Urgency and variants in the *DOCK1* gene on chromosome 10 (lead SNP rs59879440; *p* = 1.26 × 10^−7^; MAF = 0.1); and between Lack of Premeditation and rs6928101 (*p* = 2.7 × 10^−7^; MAF = 0.49) in the *KCNQ5* gene on chromosome 6, and rs17292179 (*p* = 4.2 × 10^−7^; MAF = 0.06) near the *CADM2* gene on chromosome 3 (but not in LD with previously reported *CADM2* SNPs). In particular, the *STK17B*–*HECW2* gene region (Figure S8) contains several genome‐wide significant hits (in moderate LD with the lead SNP identified in ABCD data; *R* ^2^ = 0.36–0.37) from published GWASs of smoking initiation and brain phenotypes, such as cortical thickness and surface area. The MAGMA analysis did not identify any gene‐based association (Table S8).

### 3.4 Polygenic Score Analysis

**FIGURE 2:**
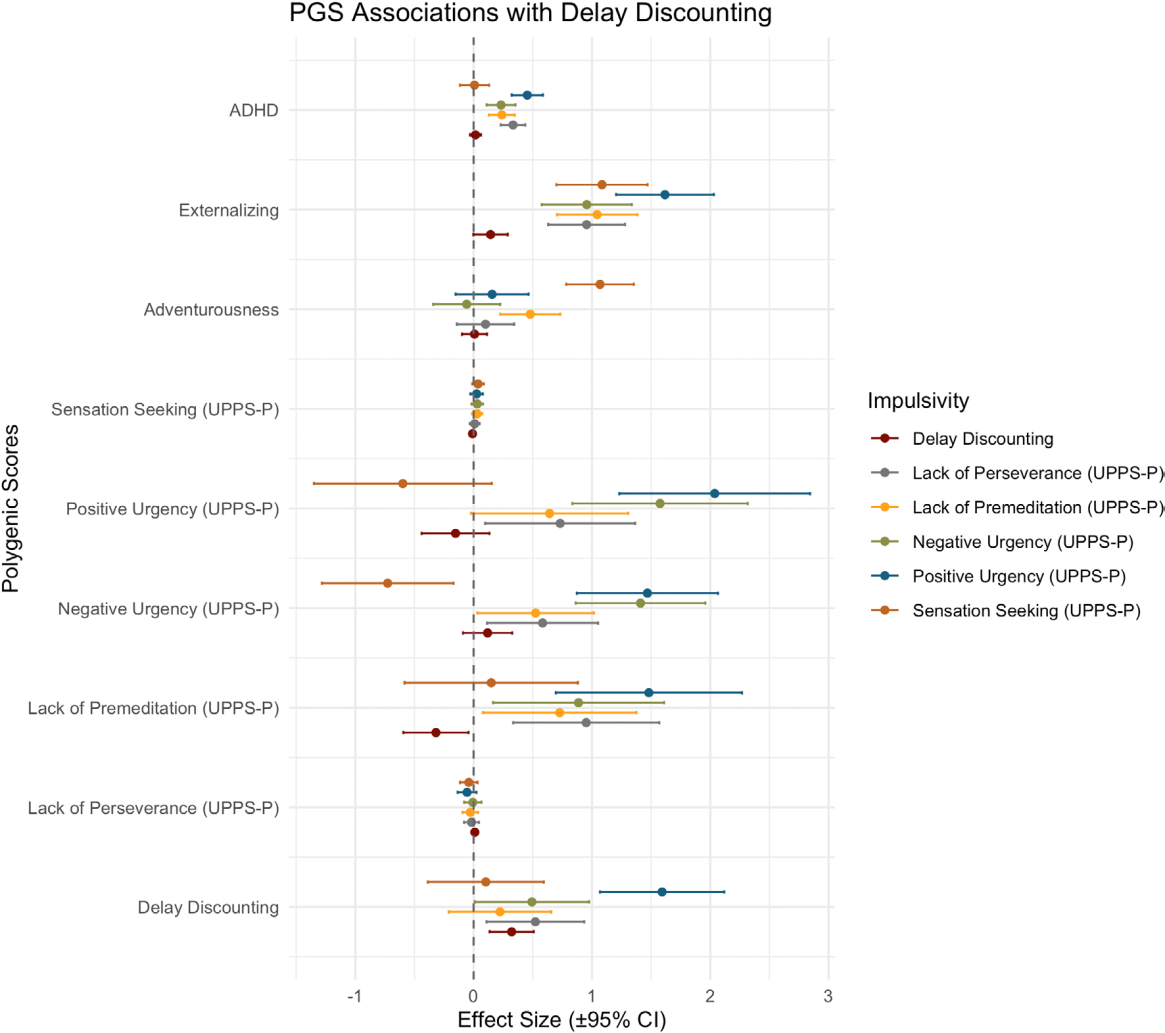
PGSs association with impulsivity traits. The *y*‐axis reflects standardized regression coefficients (i.e., effect sizes), and error bars denote 95% confidence intervals. Each point represents the association between a given PGS and an impulsivity phenotype—delay discounting and the five UPPS‐P subscales—estimated using linear regression. PGS‐impulsivity associations are staggered by trait within each PGS group. The dashed horizontal line indicates the null (zero effect).

DD and the UPPS‐P urgencies PGSs derived using adult GWASs were associated with their corresponding phenotypes in children (Table S9). Trait‐specific PGSs explained 0.36%–0.44% of the phenotypic variance (*p* = 7.5 × 10^−4^–4.7 × 10^−7^). While the PGS for Lack of Premeditation was not associated with the trait itself, it was significantly associated with both urgency indicators (0.09% and 0.22%, *p* = 0.016 and 2.3 × 10^−4^) and Lack of Perseverance (0.15%, *p* = 2.5 × 10^−3^). The externalizing PGS was broadly associated with all UPPS‐P indices (adjusted *R* ^2^ = 0.42%–1.02%; *p* = 8.5 × 10^−7^–1.9 × 10^−14^), but not with DD (Figure 2). Similarly, the ADHD PGS was also associated with all UPPS‐P indices (adjusted *R* ^2^ = 0.23%–0.79%; *p* = 1.9 × 10^−4^–1.8 × 10^−11^), except Sensation Seeking, which was strongly associated with the adventurousness PGS (adjusted *R* ^2^ = 0.93%; *p* = 2.6 × 10^−13^). Except for DD, all impulsivity measures showed improved variance explained when incorporating additional PGSs, with a significant non‐overlapping contribution from the externalizing PGS beyond trait‐specific predictors (Figure S9).

## 4 Discussion

This study represents the first GWAS of impulsivity phenotypes in children. We observed significant heritability for DD and Positive Urgency in children from the ABCD study, supporting the role of genetic influences on these traits early in development. Moreover, for the first time, we confirmed a broad overlap in genetic architecture between child and adult traits for DD and both urgency indicators, suggesting at least partial continuity in the underlying genetics across developmental stages.

While we confirmed partial genetic overlap between children and adult impulsivity for some, however, the remaining impulsivity indicators were not associated with their PGSs. This was surprising as we had successfully replicated these associations in adult populations [7, 77]. In the case of Lack of Premeditation, it was possibly due to the smaller sample size of ABCD children, which could limit the power of the analysis to surpass the FDR correction. In the other case, we hypothesize that a lack of association could reflect the early, undifferentiated nature of these traits, which may separate into more distinct facets of impulsivity with age. For instance, Sensation Seeking was significantly associated with other related PGSs derived in adults (e.g., adventurousness and externalizing) but not the trait PGS itself. Further, it has poor internal consistency as compared to the other UPPS‐P indicators in ABCD was also noted [87].

More broadly, our results are in line with other studies using adult‐derived PGS in the ABCD cohort. We confirmed the associations between externalizing and the majority of impulsivity phenotypes found in another study [88]. Our results also confirmed a similar lack of associations between PGS for externalizing behaviors and DD [89]. Interestingly, the ADHD PGS was also associated with multiple UPPS‐P phenotypes measured in children, suggesting different facets of childhood impulsivity tapping into the genetic basis of inattention and hyperactivity that are signatures of ADHD at both early adolescence and adulthood [90], which may become more pronounced over time [91]. These polygenic overlaps and shared liability across behavioral traits were supported by the observation that in addition to trait‐specific PGS, related PGSs also contributed significantly to trait variance in ABCD for all UPPS‐P indicators (Figure S9). This suggests that while the genetics of related traits and impulsivity overlap, some facets of childhood impulsivity (e.g., Sensation Seeking and Lack of Perseverance) may be less aligned with their corresponding adult traits, potentially reflecting developmental or context‐dependent differences in their manifestation.

Finally, DD had a few sub‐genome‐wide associations with several genetic variants in *HECW2*, but these associations were not observed in *prior* GWASs of DD in adults. The *HECW2* gene has key roles in a number of neurodevelopmental processes, such as the proliferation, migration, and differentiation of neural crest cells that regulate the glial cell line‐derived neurotrophic factor (GDNF)/Ret signaling [92, 93]. Rare mutations in this gene have also been robustly linked to neurodevelopmental delay, epilepsy, behavioral abnormalities, and autistic spectrum disorders [94, 95, 96, 97, 98], which are also characterized by elevated impulsivity [99, 100, 101, 102], but not with DD specifically. The other interesting finding was the replication of variants near the *CADM2* gene with Lack of Premeditation that was previously reported in adults [39].

Several limitations bear mentioning. A primary limitation of the present GWAS is the modest sample size, which limits statistical power for genome‐wide discovery, which was further exacerbated by the loss of samples due to the need for aggressive QC of the DD phenotype. While the ABCD cohort is the largest sample available for these traits in childhood, these findings remain underpowered compared with adult GWASs for similar phenotypes. In particular, our results suggest moderate heritability for UPPS‐P indicators (6%–10%), similar to values reported in adult populations. To achieve > 80% power to confirm these estimates, a sample size of at least *n* =9000 to 15,000 would be required. Additionally, although the ABCD study is more diverse than many previous large‐scale behavioral genetics cohorts, multi‐ancestry GWASs still require larger sub‐sample sizes to yield ancestry‐specific discoveries and ensure generalizability of PGS across populations. Finally, while the psychometric properties of the UPPS‐P Impulsive Behavior Scale have been established in ABCD [103], data quality of DD has been problematic in terms of (1) the most appropriate method to generate summary measure of DD [104, 105], (2) low effort/low attention performance can violate assumptions of the test [105].

To conclude, these results reveal, for the first time, the molecular genetic correlates of diverse impulsivity phenotypes in children, pointing to additive genetic influences and difference in genetic architecture compared with adults. Future longitudinal genetic studies, as have recently been undertaken in adults [77], are warranted to determine whether these genetic influences remain stable or change across development, providing a true life‐course perspective. Integrating genetic data from children in longitudinal cohorts will permit tracking changes in impulsivity over time, revealing the interplay between genetic and environmental factors during early development when children and their brains are both malleable, but also vulnerable.

## Conflicts of Interest

The authors declare no conflicts of interest.

## Supporting information


**Data S1:** Supporting Information.

## Data Availability

Genotype and phenotype data of ABCD Data Release 5.0 are available through the NIMH Data Archive pending a successful application. The PGS analyses included summary statistics from published GWASs made available by the Psychiatric Genomics Consortium (PGC), the UK Biobank, the Externalizing Consortium, and 23andMe Inc. Due to concern for participants' privacy, the full summary statistics of delay discounting and the five UPPS‐P subscales are available for academic use upon request from 23andMe. The full summary statistics based on ABCD data will be made available via GWAS catalog (https://www.ebi.ac.uk/gwas/).

## References

[1] J. M. Berg , R. D. Latzman , N. G. Bliwise , and S. O. Lilienfeld , “Parsing the Heterogeneity of Impulsivity: A Meta‐Analytic Review of the Behavioral Implications of the UPPS for Psychopathology,” Psychological Assessment 27, no. 4 (2015): 1129–1146, 10.1037/PAS0000111. 25822833

[2] T. E. Moffitt , L. Arseneault , D. Belsky , et al., “A Gradient of Childhood Self‐Control Predicts Health, Wealth, and Public Safety,” Proceedings of the National Academy of Sciences of the United States of America 108, no. 7 (2011): 2693–2698, 10.1073/PNAS.1010076108. 21262822 PMC3041102

[3] J. MacKillop , M. T. Amlung , L. R. Few , L. A. Ray , L. H. Sweet , and M. R. Munafò , “Delayed Reward Discounting and Addictive Behavior: A Meta‐Analysis,” Psychopharmacology 216, no. 3 (2011): 305–321, 10.1007/s00213-011-2229-0. 21373791 PMC3201846

[4] J. MacKillop , “Integrating Behavioral Economics and Behavioral Genetics: Delayed Reward Discounting as an Endophenotype for Addictive Disorders,” Journal of the Experimental Analysis of Behavior 99, no. 1 (2013): 14–31, 10.1002/jeab.4. 23344986 PMC3881595

[5] M. J. Kreek , D. A. Nielsen , E. R. Butelman , and K. S. LaForge , “Genetic Influences on Impulsivity, Risk Taking, Stress Responsivity and Vulnerability to Drug Abuse and Addiction,” Nature Neuroscience 8, no. 11 (2005): 1450–1457, 10.1038/NN1583. 16251987

[6] J. MacKillop and M. R. Munafò , Genetic Influences on Addiction: An Intermediate Phenotype Approach, ed. J. MacKillop and M. R. Munafò (MIT Press, 2013).

[7] W. Q. Deng , K. Belisario , J. C. Gray , et al., “Leveraging Related Health Phenotypes for Polygenic Prediction of Impulsive Choice, Impulsive Action, and Impulsive Personality Traits in 1534 European Ancestry Community Adults,” Genes, Brain, and Behavior 22, no. 3 (2023): e12848, 10.1111/GBB.12848. 37060189 PMC10242187

[8] J. MacKillop , J. Weafer , J. Gray , A. Oshri , A. Palmer , and H. de Wit , “The Latent Structure of Impulsivity: Impulsive Choice, Impulsive Action, and Impulsive Personality Traits,” Psychopharmacology 233, no. 18 (2016): 3361–3370, 10.1007/S00213-016-4372-0. 27449350 PMC5204128

[9] K. Kozak , A. M. Lucatch , D. J. E. Lowe , I. M. Balodis , J. MacKillop , and T. P. George , “The Neurobiology of Impulsivity and Substance Use Disorders: Implications for Treatment,” Annals of the New York Academy of Sciences 1451, no. 1 (2019): 71–91, 10.1111/nyas.13977. 30291624 PMC6450787

[10] A. Verdejo‐García , A. J. Lawrence , and L. Clark , “Impulsivity as a Vulnerability Marker for Substance‐Use Disorders: Review of Findings From High‐Risk Research, Problem Gamblers and Genetic Association Studies,” Neuroscience and Biobehavioral Reviews 32, no. 4 (2008): 777–810, 10.1016/j.neubiorev.2007.11.003. 18295884

[11] J. E. Mazur , “An Adjusting Procedure for Studying Delayed Reinforcement,” in, ed. M. L. Commons , J. E. Mazur , J. A. Nevin , and H. Rachlin (1987).

[12] S. P. Whiteside and D. R. Lynam , “The Five Factor Model and Impulsivity: Using a Structural Model of Personality to Understand Impulsivity,” Personality and Individual Differences 30, no. 4 (2001): 669–689, 10.1016/S0191-8869(00)00064-7.

[13] M. A. Cyders , A. K. Littlefield , S. Coffey , and K. A. Karyadi , “Examination of a Short English Version of the UPPS‐P Impulsive Behavior Scale,” Addictive Behaviors 39, no. 9 (2014): 1372–1376, 10.1016/j.addbeh.2014.02.013. 24636739 PMC4055534

[14] N. Castellanos‐Ryan , F. N. Briere , M. O'Leary‐Barrett , et al., “The Structure of Psychopathology in Adolescence and Its Common Personality and Cognitive Correlates,” Journal of Abnormal Psychology 125, no. 8 (2016): 1039–1052, 10.1037/ABN0000193. 27819466 PMC5098414

[15] C. Sheffer , J. MacKillop , J. McGeary , et al., “Delay Discounting, Locus of Control, and Cognitive Impulsiveness Independently Predict Tobacco Dependence Treatment Outcomes in a Highly Dependent, Lower Socioeconomic Group of Smokers,” American Journal on Addictions 21, no. 3 (2012): 221–232, 10.1111/j.1521-0391.2012.00224.x. 22494224 PMC3567840

[16] R. J. Green , B. J. Wolf , A. Chen , et al., “Predictors of Substance Use Initiation by Early Adolescence,” American Journal of Psychiatry 181, no. 5 (2024): 423–433, 10.1176/APPI.AJP.20230882. 38706327 PMC11411615

[17] N. Doran , R. Khoddam , P. E. Sanders , C. A. Schweizer , R. S. Trim , and M. G. Myers , “A Prospective Study of the Acquired Preparedness Model: The Effects of Impulsivity and Expectancies on Smoking Initiation in College Students,” Psychology of Addictive Behaviors 27, no. 3 (2013): 714–722, 10.1037/a0028988. 22686965 PMC4779050

[18] G. T. Smith and M. A. Cyders , “Integrating Affect and Impulsivity: The Role of Positive and Negative Urgency in Substance Use Risk,” Drug and Alcohol Dependence 163 (2016): S3–S12, 10.1016/J.DRUGALCDEP.2015.08.038. 27306729 PMC4911536

[19] A. P. Anokhin , S. Golosheykin , J. D. Grant , and A. C. Heath , “Heritability of Delay Discounting in Adolescence: A Longitudinal Twin Study,” Behavior Genetics 41, no. 2 (2011): 175–183, 10.1007/s10519-010-9384-7. 20700643 PMC3036802

[20] A. P. Anokhin , J. D. Grant , R. C. Mulligan , and A. C. Heath , “The Genetics of Impulsivity: Evidence for the Heritability of Delay Discounting,” Biological Psychiatry 77, no. 10 (2015): 887–894, 10.1016/j.biopsych.2014.10.022. 25555481 PMC4416979

[21] S. Niv , C. Tuvblad , A. Raine , P. Wang , and L. A. Baker , “Heritability and Longitudinal Stability of Impulsivity in Adolescence,” Behavior Genetics 42, no. 3 (2012): 378–392, 10.1007/s10519-011-9518-6. 22089391 PMC3351554

[22] J. Tiego , S. R. Chamberlain , B. J. Harrison , et al., “Heritability of Overlapping Impulsivity and Compulsivity Dimensional Phenotypes,” Scientific Reports 10, no. 1 (2020): 14378, 10.1038/s41598-020-71013-x. 32873811 PMC7463011

[23] R. W. Hook , J. E. Grant , K. Ioannidis , et al., “Trans‐Diagnostic Measurement of Impulsivity and Compulsivity: A Review of Self‐Report Tools,” Neuroscience and Biobehavioral Reviews 120 (2021): 455–469, 10.1016/J.NEUBIOREV.2020.10.007. 33115636 PMC7116678

[24] E. E. Levitt , A. Oshri , M. Amlung , et al., “Evaluation of Delay Discounting as a Transdiagnostic Research Domain Criteria Indicator in 1388 General Community Adults,” Psychological Medicine 53, no. 4 (2023): 1649–1657, 10.1017/S0033291721005110. 35080193 PMC10009385

[25] D. E. Gustavson , N. P. Friedman , P. Fontanillas , S. L. Elson , A. A. Palmer , and S. Sanchez‐Roige , “The Latent Genetic Structure of Impulsivity and Its Relation to Internalizing Psychopathology,” Psychological Science 31, no. 8 (2020): 1025–1035, 10.1177/0956797620938160. 32716714 PMC7427138

[26] L. Vilar‐Ribó , A. S. Hatoum , and A. D. Grotzinger , et al., “Impulsivity Facets and Substance Use Involvement: Insights From Genomic Structural Equation Modeling,” Psychological Medicine 55 (2025): e51, 10.1017/S0033291725000145. 39957498 PMC12039315

[27] A. S. Hatoum , S. M. C. Colbert , E. C. Johnson , et al., “Multivariate Genome‐Wide Association Meta‐Analysis of Over 1 Million Subjects Identifies Loci Underlying Multiple Substance Use Disorders,” Nature Mental Health 1, no. 3 (2023): 210–223, 10.1038/s44220-023-00034-y. 37250466 PMC10217792

[28] G. R. B. Saunders , X. Wang , F. Chen , et al., “Genetic Diversity Fuels Gene Discovery for Tobacco and Alcohol Use,” Nature 612, no. 7941 (2022): 720–724, 10.1038/s41586-022-05477-4. 36477530 PMC9771818

[29] Y. Cheng , C. Dao , H. Zhou , et al., “Multi‐Trait Genome‐Wide Association Analyses Leveraging Alcohol Use Disorder Findings Identify Novel Loci for Smoking Behaviors in the Million Veteran Program,” Translational Psychiatry 13, no. 1 (2023): 148, 10.1038/s41398-023-02409-2. 37147289 PMC10162964

[30] K. Xu , B. Li , K. A. McGinnis , et al., “Genome‐Wide Association Study of Smoking Trajectory and Meta‐Analysis of Smoking Status in 842,000 Individuals,” Nature Communications 11, no. 1 (2020): 5302, 10.1038/S41467-020-18489-3. PMC7598939 33082346

[31] H. R. Kranzler , H. Zhou , R. L. Kember , et al., “Genome‐Wide Association Study of Alcohol Consumption and Use Disorder in 274,424 Individuals From Multiple Populations,” Nature Communications 10, no. 1 (2019): 1499, 10.1038/s41467-019-09480-8. PMC6445072 30940813

[32] J. D. Deak , H. Zhou , M. Galimberti , et al., “Genome‐Wide Association Study in Individuals of European and African Ancestry and Multi‐Trait Analysis of Opioid Use Disorder Identifies 19 Independent Genome‐Wide Significant Risk Loci,” Molecular Psychiatry 27, no. 10 (2022): 3970–3979, 10.1038/s41380-022-01709-1. 35879402 PMC9718667

[33] H. Zhou , J. M. Sealock , S. Sanchez‐Roige , et al., “Genome‐Wide Meta‐Analysis of Problematic Alcohol Use in 435,563 Individuals Yields Insights Into Biology and Relationships With Other Traits,” Nature Neuroscience 23, no. 7 (2020): 809–818, 10.1038/S41593-020-0643-5. 32451486 PMC7485556

[34] D. F. Levey , M. Galimberti , J. D. Deak , et al., “Multi‐Ancestry Genome‐Wide Association Study of Cannabis Use Disorder Yields Insight Into Disease Biology and Public Health Implications,” Nature Genetics 55, no. 12 (2023): 2094–2103, 10.1038/s41588-023-01563-z. 37985822 PMC10703690

[35] H. Zhou , R. L. Kember , J. D. Deak , et al., “Multi‐Ancestry Study of the Genetics of Problematic Alcohol Use in Over 1 Million Individuals,” Nature Medicine 29, no. 12 (2023): 3184–3192, 10.1038/s41591-023-02653-5. PMC10719093 38062264

[36] J. MacKillop , J. C. Gray , J. Weafer , S. Sanchez‐Roige , A. A. Palmer , and H. De Wit , “Genetic Influences on Delayed Reward Discounting: A Genome‐Wide Prioritized Subset Approach,” Experimental and Clinical Psychopharmacology 27, no. 1 (2019): 29–37, 10.1037/pha0000227. 30265060 PMC6908809

[37] S. Sanchez‐Roige , P. Fontanillas , S. L. Elson , et al., “Genome‐Wide Association Studies of Impulsive Personality Traits (BIS‐11 and UPPS‐P) and Drug Experimentation in up to 22,861 Adult Research Participants Identify Loci in the CACNA1I and CADM2 Genes,” Journal of Neuroscience 39, no. 13 (2019): 2562–2572, 10.1523/JNEUROSCI.2662-18.2019. 30718321 PMC6435820

[38] S. Sanchez‐Roige , P. Fontanillas , S. L. Elson , et al., “Genome‐Wide Association Study of Delay Discounting in 23,217 Adult Research Participants of European Ancestry,” Nature Neuroscience 21, no. 1 (2018): 16–18, 10.1038/s41593-017-0032-x. 29230059 PMC6984001

[39] S. Sanchez‐Roige , M. V. Jennings , H. H. A. Thorpe , et al., “CADM2 is Implicated in Impulsive Personality and Numerous Other Traits by Genome‐ and Phenome‐Wide Association Studies in Humans and Mice,” Translational Psychiatry 13, no. 1 (2023): 1–11, 10.1038/s41398-023-02453-y. 37173343 PMC10182097

[40] H. H. Thorpe , R. B. Cupertino , S. R. Pakala , et al., “Genome‐Wide Association Study of Delay Discounting in 134,935 Individuals Identifies Novel Loci and Transdiagnostic Associations Across Mental and Physical Health,” medRxiv 17 (2024): 24314244, 10.1101/2024.09.27.24314244.

[41] R. Karlsson Linnér , T. T. Mallard , P. B. Barr , et al., “Multivariate Analysis of 1.5 Million People Identifies Genetic Associations With Traits Related to Self‐Regulation and Addiction,” Nature Neuroscience 24, no. 10 (2021): 1367–1376, 10.1038/s41593-021-00908-3. 34446935 PMC8484054

[42] D. E. Gustavson , C. L. Morrison , T. T. Mallard , et al., “Executive Function and Impulsivity Predict Distinct Genetic Variance in Internalizing Problems, Externalizing Problems, Thought Disorders, and Compulsive Disorders: A Genomic Structural Equation Modeling Study,” Clinical Psychological Science 12, no. 5 (2024): 865–881, 10.1177/21677026231207845. 39323941 PMC11423426

[43] L. Vilar‐Ribó , A. S. Hatoum , A. D. Grotzinger , et al., “Impulsivity Facets and Substance Use Involvement: Insights From Genomic Structural Equation Modeling,” Psychological Medicine 55 (2025): e51, 10.1017/S0033291725000145. 39957498 PMC12039315

[44] J. H. Patton , M. S. Stanford , and E. S. Barratt , “Factor Structure of the Barratt Impulsiveness Scale,” Journal of Clinical Psychology 51, no. 6 (1995): 768–774, 10.1002/1097-4679(199511)51:6<768::AID-JCLP2270510607>3.0.CO;2–1. 8778124

[45] M. Geurten , C. Catale , P. Gay , S. Deplus , and J. Billieux , “Measuring Impulsivity in Children: Adaptation and Validation of a Short Version of the UPPS‐P Impulsive Behaviors Scale in Children and Investigation of Its Links With ADHD,” Journal of Attention Disorders 25, no. 1 (2021): 105–114, 10.1177/1087054718775831. 29771172

[46] P. Burns , O. Fay , M. F. McCafferty , V. McKeever , C. Atance , and T. McCormack , “Examining Children's Ability to Delay Reward: Is the Delay Discounting Task a Suitable Measure?,” Journal of Behavioral Decision Making 33, no. 2 (2020): 208–219, 10.1002/BDM.2154.

[47] S. D. Klein , P. F. Collins , and M. Luciana , “Developmental Trajectories of Delay Discounting From Childhood to Young Adulthood: Longitudinal Associations and Test‐Retest Reliability,” Cognitive Psychology 139 (2022): 101518, 10.1016/J.COGPSYCH.2022.101518. 36183669 PMC10888509

[48] R. J. Kohler , S. D. Lichenstein , and S. W. Yip , “Hyperbolic Discounting Rates and Risk for Problematic Alcohol Use in Youth Enrolled in the Adolescent Brain and Cognitive Development Study,” Addiction Biology 27, no. 2 (2022): e13160, 10.1111/ADB.13160. 35229959 PMC9289942

[49] M. E. Sloan , M. Sanches , J. Tanabe , and J. L. Gowin , “Delay Discounting and Family History of Psychopathology in Children Ages 9–11,” Scientific Reports 13, no. 1 (2023): 1–9, 10.1038/s41598-023-49148-4. 38081908 PMC10713649

[50] D. Romer , “Adolescent Risk Taking, Impulsivity, and Brain Development: Implications for Prevention,” Developmental Psychobiology 52, no. 3 (2010): 263–276, 10.1002/dev.20442. 20175097 PMC3445337

[51] G. M. Rosenbaum and C. A. Hartley , “Developmental Perspectives on Risky and Impulsive Choice,” Philosophical Transactions of the Royal Society, B: Biological Sciences 374, no. 1766 (2019): 20180133, 10.1098/rstb.2018.0133. PMC6335462 30966918

[52] M. Haas , A. Hiemisch , M. Vogel , O. Wagner , W. Kiess , and T. Poulain , “Sensation Seeking in 3‐ to 6‐Year‐Old Children: Associations With Socio‐Demographic Parameters and Behavioural Difficulties,” BMC Pediatrics 19, no. 1 (2019): 77, 10.1186/s12887-019-1450-6. 30857528 PMC6410503

[53] F. Vergunst , M. Commisso , M. C. Geoffroy , et al., “Association of Childhood Externalizing, Internalizing, and Comorbid Symptoms With Long‐Term Economic and Social Outcomes,” JAMA Network Open 6, no. 1 (2023): e2249568, 10.1001/JAMANETWORKOPEN.2022.49568. 36622675 PMC9856729

[54] M. Weeks , G. B. Ploubidis , J. Cairney , T. C. Wild , K. Naicker , and I. Colman , “Developmental Pathways Linking Childhood and Adolescent Internalizing, Externalizing, Academic Competence, and Adolescent Depression,” Journal of Adolescence 51 (2016): 30–40, 10.1016/j.adolescence.2016.05.009. 27288965

[55] T. P. Beauchaine , A. R. Zisner , and C. L. Sauder , “Trait Impulsivity and the Externalizing Spectrum,” Annual Review of Clinical Psychology 13 (2017): 343–368, 10.1146/annurev-clinpsy-021815-093253. 28375718

[56] S. Bezdjian , L. A. Baker , and C. Tuvblad , “Genetic and Environmental Influences on Impulsivity: A Meta‐Analysis of Twin, Family and Adoption Studies,” Clinical Psychology Review 31, no. 7 (2011): 1209–1223, 10.1016/J.CPR.2011.07.005. 21889436 PMC3176916

[57] H. Shimelis , M. T. Oetjens , L. K. Walsh , et al., “Prevalence and Penetrance of Rare Pathogenic Variants in Neurodevelopmental Psychiatric Genes in a Health Care System Population,” American Journal of Psychiatry 180, no. 1 (2023): 65–72, 10.1176/APPI.AJP.22010062. 36475376 PMC10017070

[58] M. Nakanishi , M. P. Anderson , and T. Takumi , “Recent Genetic and Functional Insights in Autism Spectrum Disorder,” Current Opinion in Neurology 32, no. 4 (2019): 627–634, 10.1097/WCO.0000000000000718. 31135459 PMC6959126

[59] E. L. Casanova , J. L. Sharp , H. Chakraborty , N. S. Sumi , and M. F. Casanova , “Genes With High Penetrance for Syndromic and Non‐Syndromic Autism Typically Function Within the Nucleus and Regulate Gene Expression,” Molecular Autism 7 (2016): 18, 10.1186/S13229-016-0082-Z. 26985359 PMC4793536

[60] R. Leshem , “Brain Development, Impulsivity, Risky Decision Making, and Cognitive Control: Integrating Cognitive and Socioemotional Processes During Adolescence—An Introduction to the Special Issue,” Developmental Neuropsychology 41, no. 1–2 (2016): 1–5, 10.1080/87565641.2016.1187033. 27392088

[61] N. D. Volkow , G. F. Koob , R. T. Croyle , et al., “The Conception of the ABCD Study: From Substance Use to a Broad NIH Collaboration,” Developmental Cognitive Neuroscience 32 (2018): 4–7, 10.1016/j.dcn.2017.10.002. 29051027 PMC5893417

[62] H. Garavan , H. Bartsch , K. Conway , et al., “Recruiting the ABCD Sample: Design Considerations and Procedures,” Developmental Cognitive Neuroscience 32 (2018): 16–22, 10.1016/j.dcn.2018.04.004. 29703560 PMC6314286

[63] D. M. Barch , M. D. Albaugh , S. Avenevoli , et al., “Demographic, Physical and Mental Health Assessments in the Adolescent Brain and Cognitive Development Study: Rationale and Description,” Developmental Cognitive Neuroscience 32 (2018): 55–66, 10.1016/j.dcn.2017.10.010. 29113758 PMC5934320

[64] K. A. Uban , M. K. Horton , J. Jacobus , et al., “Biospecimens and the ABCD Study: Rationale, Methods of Collection, Measurement and Early Data,” Developmental Cognitive Neuroscience 32 (2018): 97–106, 10.1016/j.dcn.2018.03.005. 29606560 PMC6487488

[65] B. J. Casey , T. Cannonier , M. I. Conley , et al., “The Adolescent Brain Cognitive Development (ABCD) Study: Imaging Acquisition Across 21 Sites,” Developmental Cognitive Neuroscience 32 (2018): 43–54, 10.1016/j.dcn.2018.03.001. 29567376 PMC5999559

[66] M. Luciana , J. M. Bjork , B. J. Nagel , et al., “Adolescent Neurocognitive Development and Impacts of Substance Use: Overview of the Adolescent Brain Cognitive Development (ABCD) Baseline Neurocognition Battery,” Developmental Cognitive Neuroscience 32 (2018): 67–79, 10.1016/J.DCN.2018.02.006. 29525452 PMC6039970

[67] M. N. Koffarnus and W. K. Bickel , “A 5‐Trial Adjusting Delay Discounting Task: Accurate Discount Rates in Less Than One Minute,” Experimental and Clinical Psychopharmacology 22, no. 3 (2014): 222–228, 10.1037/a0035973. 24708144 PMC4461028

[68] M. Elsayed , M. M. Owens , I. Balodis , and J. MacKillop , “Empirical Examination of Working Memory Performance and Its Neural Correlates in Relation to Delay Discounting in Two Large Samples,” Behavioural Brain Research 475 (2024): 115217, 10.1016/J.BBR.2024.115217. 39181217

[69] T. J. White , R. Redner , J. M. Skelly , and S. T. Higgins , “Examination of a Recommended Algorithm for Eliminating Nonsystematic Delay Discounting Response Sets,” Drug and Alcohol Dependence 154 (2015): 300–303, 10.1016/J.DRUGALCDEP.2015.07.011. 26208791 PMC4752816

[70] J. W. Baurley , C. K. Edlund , C. I. Pardamean , D. V. Conti , and A. W. Bergen , “Smokescreen: A Targeted Genotyping Array for Addiction Research,” BMC Genomics 17, no. 1 (2016): 145, 10.1186/s12864-016-2495-7. 26921259 PMC4769529

[71] A. Auton , G. R. Abecasis , D. M. Altshuler , et al., “A Global Reference for Human Genetic Variation,” Nature 526, no. 7571 (2015): 68–74, 10.1038/nature15393. 26432245 PMC4750478

[72] B. Zhao , X. Yang , and H. Zhu , “Estimating Trans‐Ancestry Genetic Correlation With Unbalanced Data Resources,” Journal of the American Statistical Association 119, no. 546 (2024): 839–850, 10.1080/01621459.2024.2344703. 39219674 PMC11364214

[73] Y. Ding , K. Hou , Z. Xu , et al., “Polygenic Scoring Accuracy Varies Across the Genetic Ancestry Continuum,” Nature 618, no. 7966 (2023): 774–781, 10.1038/s41586-023-06079-4. 37198491 PMC10284707

[74] J. Yang , S. H. Lee , N. R. Wray , M. E. Goddard , and P. M. Visscher , “GCTA‐GREML Accounts for Linkage Disequilibrium When Estimating Genetic Variance From Genome‐Wide SNPs,” Proceedings of the National Academy of Sciences of the United States of America 113, no. 32 (2016): 201602743, 10.1073/pnas.1602743113. PMC4987770 27457963

[75] B. Bulik‐Sullivan , P. R. Loh , H. K. Finucane , et al., “LD Score Regression Distinguishes Confounding From Polygenicity in Genome‐Wide Association Studies,” Nature Genetics 47, no. 3 (2015): 291–295, 10.1038/NG.3211. 25642630 PMC4495769

[76] Y. Zhang , Y. Cheng , W. Jiang , Y. Ye , Q. Lu , and H. Zhao , “Comparison of Methods for Estimating Genetic Correlation Between Complex Traits Using GWAS Summary Statistics,” Briefings in Bioinformatics 22, no. 5 (2021): bbaa442, 10.1093/bib/bbaa442. 33497438 PMC8425307

[77] W. Q. Deng , K. Belisario , M. R. Munafò , and J. MacKillop , “Longitudinal Characterization of Impulsivity Phenotypes Boosts Signal for Genomic Correlates and Heritability,” Molecular Psychiatry 30, no. 2 (2024): 1–618, 10.1038/s41380-024-02704-4. 39181994

[78] C. C. Chang , C. C. Chow , L. C. A. M. Tellier , S. Vattikuti , S. M. Purcell , and J. J. Lee , “Second‐Generation PLINK: Rising to the Challenge of Larger and Richer Datasets,” GigaScience 4, no. 1 (2015): 7, 10.1186/s13742-015-0047-8. 25722852 PMC4342193

[79] R. J. Pruim , R. P. Welch , S. Sanna , et al., “LocusZoom: Regional Visualization of Genome‐Wide Association Scan Results,” Bioinformatics 26, no. 18 (2010): 2336–2337, 10.1093/BIOINFORMATICS/BTQ419. 20634204 PMC2935401

[80] A. P. Boughton , R. P. Welch , M. Flickinger , et al., “LocusZoom.Js: Interactive and Embeddable Visualization of Genetic Association Study Results,” Bioinformatics 37, no. 18 (2021): 3017–3018, 10.1093/bioinformatics/btab186. 33734315 PMC8479674

[81] B. Chen , R. V. Craiu , L. J. Strug , and L. Sun , “The X Factor: A Robust and Powerful Approach to X‐Chromosome‐Inclusive Whole‐Genome Association Studies,” Genetic Epidemiology 45, no. 7 (2021): 694–709, 10.1002/gepi.22422. 34224641 PMC9292551

[82] C. A. de Leeuw , J. M. Mooij , T. Heskes , and D. Posthuma , “MAGMA: Generalized Gene‐Set Analysis of GWAS Data,” PLoS Computational Biology 11, no. 4 (2015): e1004219, 10.1371/journal.pcbi.1004219. 25885710 PMC4401657

[83] F. Privé , J. Arbel , and B. J. Vilhjálmsson , “LDpred2: Better, Faster, Stronger,” Bioinformatics 36, no. 22–23 (2020): 5424–5431, 10.1093/bioinformatics/btaa1029. PMC8016455 33326037

[84] R. Karlsson Linnér , P. Biroli , E. Kong , et al., “Genome‐Wide Association Analyses of Risk Tolerance and Risky Behaviors in Over 1 Million Individuals Identify Hundreds of Loci and Shared Genetic Influences,” Nature Genetics 51, no. 2 (2019): 245–257, 10.1038/s41588-018-0309-3. 30643258 PMC6713272

[85] D. Demontis , G. B. Walters , G. Athanasiadis , et al., “Genome‐Wide Analyses of ADHD Identify 27 Risk Loci, Refine the Genetic Architecture and Implicate Several Cognitive Domains,” Nature Genetics 55, no. 2 (2023): 198–208, 10.1038/s41588-022-01285-8. 36702997 PMC10914347

[86] R Core Team , R: A Language and Environment for Statistical Computing (R Foundation for Statistical Computing, 2021), http://www.R‐project.org. R‐project org.

[87] D. M. Barch , M. D. Albaugh , S. Avenevoli , et al., “Demographic, Physical and Mental Health Assessments in the Adolescent Brain and Cognitive Development Study: Rationale and Description,” Developmental Cognitive Neuroscience 32 (2017): 55, 10.1016/J.DCN.2017.10.010. 29113758 PMC5934320

[88] J. Teeuw , N. R. Mota , M. Klein , et al., “Polygenic Risk Scores and Brain Structures Both Contribute to Externalizing Behavior in Childhood ‐ A Study in the Adolescent Brain and Cognitive Development (ABCD) Cohort,” Neuroscience Applied 2 (2023): 101128, 10.1016/j.nsa.2023.101128. 40655980 PMC12244044

[89] J. A. Rabinowitz , N. Thomas , J. C. Strickland , et al., “Genetic Propensity for Delay Discounting and Educational Attainment in Adults Are Associated With Delay Discounting in Preadolescents: Findings From the Adolescent Brain Cognitive Development Study,” Genes, Brain, and Behavior 24, no. 2 (2025): e70020, 10.1111/GBB.70020. 40147852 PMC11949538

[90] T. J. Spencer , J. Biederman , and E. Mick , “Attention‐Deficit/Hyperactivity Disorder: Diagnosis, Lifespan, Comorbidities, and Neurobiology,” Ambulatory Pediatrics 7, no. 1 Suppl (2007): 73–81, 10.1016/J.AMBP.2006.07.006. 17261486

[91] H. Larsson , R. Dilshad , P. Lichtenstein , and E. D. Barker , “Developmental Trajectories of DSM‐IV Symptoms of Attention‐Deficit/Hyperactivity Disorder: Genetic Effects, Family Risk and Associated Psychopathology,” Journal of Child Psychology and Psychiatry 52, no. 9 (2011): 954–963, 10.1111/J.1469-7610.2011.02379.X. 21434915

[92] K. Miyazaki , T. Ozaki , C. Kato , et al., “A Novel HECT‐Type E3 Ubiquitin Ligase, NEDL2, Stabilizes p73 and Enhances Its Transcriptional Activity,” Biochemical and Biophysical Research Communications 308, no. 1 (2003): 106–113, 10.1016/S0006-291X(03)01347-0. 12890487

[93] R. Wei , X. Qiu , S. Wang , et al., “NEDL2 Is an Essential Regulator of Enteric Neural Development and GDNF/Ret Signaling,” Cellular Signalling 27, no. 3 (2015): 578–586, 10.1016/j.cellsig.2014.12.013. 25555806

[94] E. R. Berko , M. T. Cho , C. Eng , et al., “De Novo Missense Variants in HECW2 Are Associated With Neurodevelopmental Delay and Hypotonia,” Journal of Medical Genetics 54, no. 2 (2017): 84–86, 10.1136/jmedgenet-2016-103943. 27389779 PMC5222737

[95] A. M. Krami , A. Bouzidi , M. Charif , et al., “A Homozygous Nonsense HECW2 Variant Is Associated With Neurodevelopmental Delay and Intellectual Disability,” European Journal of Medical Genetics 65, no. 6 (2022): 104515, 10.1016/j.ejmg.2022.104515. 35487419

[96] N. L. Ullman , C. L. Smith‐Hicks , S. Desai , and C. E. Stafstrom , “De Novo HECW2 Mutation Associated With Epilepsy, Developmental Decline, and Intellectual Disability: Case Report and Review of Literature,” Pediatric Neurology 85 (2018): 76–78, 10.1016/j.pediatrneurol.2018.03.005. 29807643

[97] E. C. Heide , O. Puk , S. Biskup , et al., “A Novel Likely Pathogenic Heterozygous HECW2 Missense Variant in a Family With Variable Expressivity of Neurodevelopmental Delay, Hypotonia, and Epileptiform EEG Patterns,” American Journal of Medical Genetics. Part A 185, no. 12 (2021): 3838–3843, 10.1002/ajmg.a.62427. 34327820

[98] J. Halvardson , J. J. Zhao , A. Zaghlool , et al., “Mutations in HECW2 Are Associated With Intellectual Disability and Epilepsy,” Journal of Medical Genetics 53, no. 10 (2016): 697–704, 10.1136/jmedgenet-2016-103814. 27334371 PMC5099177

[99] F. M. C. Besag , “Behavioral Aspects of Pediatric Epilepsy Syndromes,” Epilepsy & Behavior 5, no. Suppl 1 (2004): 3–13, 10.1016/j.yebeh.2003.11.002. 14725841

[100] A. Shakeshaft , N. Panjwani , R. McDowall , et al., “Trait Impulsivity in Juvenile Myoclonic Epilepsy,” Annals of Clinical Translational Neurology 8, no. 1 (2021): 138–152, 10.1002/acn3.51255. 33264519 PMC7818143

[101] A. Smith , M. Syvertsen , and D. K. Pal , “Meta‐Analysis of Response Inhibition in Juvenile Myoclonic Epilepsy,” Epilepsy & Behavior 106 (2020): 107038, 10.1016/j.yebeh.2020.107038. 32240946

[102] S. B. Messer , “Reflection‐Impulsivity: A Review,” Psychological Bulletin 83, no. 6 (1976): 1026–1052, 10.1037/0033-2909.83.6.1026.

[103] A. L. Watts , G. T. Smith , D. M. Barch , and K. J. Sher , “Factor Structure, Measurement and Structural Invariance, and External Validity of an Abbreviated Youth Version of the UPPS‐P Impulsive Behavior Scale,” Psychological Assessment 32, no. 4 (2019): 336–347, 10.1037/pas0000791. 31841018 PMC7093245

[104] J. Myerson and L. Green , “Discounting of Delayed Rewards: Models of Individual Choice,” Journal of the Experimental Analysis of Behavior 64, no. 3 (1995): 263–276, 10.1901/JEAB.1995.64-263. 16812772 PMC1350137

[105] M. W. Johnson and W. K. Bickel , “An Algorithm for Identifying Nonsystematic Delay‐Discounting Data,” Experimental and Clinical Psychopharmacology 16, no. 3 (2008): 264–274, 10.1037/1064-1297.16.3.264. 18540786 PMC2765051

