# Supplementary material for: Genome‐Wide Association Studies of Delay Discounting and Impulsive Personality Traits in Children From the Adolescent Behavior and Cognitive Development Study
Source: Genes Brain Behav. 2025 Aug 23;24(4):e70033. doi: 10.1111/gbb.70033 (PMC12374252; doi:10.1111/gbb.70033)
Supplement: Supplementary file 1 — Data S1: Supporting Information. [file GBB-24-e70033-s001.zip › gbb70033-sup-0002-FigureS1-S9@Suppl-Materials-Genomics_of_impulsivity_ABCD-revised.docx]

**Supplementary Materials for “**Genome-wide association studies of delay discounting and impulsive personality traits in children from the Adolescent Behavior and Cognitive Development Study”

Wei Q. Deng1,2,*, Mahmoud Elsayed1,2, Kyla L. Belisario1,2, Sandra Sanchez-Roige3,4,5, Abraham A. Palmer3,5, James MacKillop1,2*

1. Peter Boris Centre for Addictions Research, St. Joseph’s Healthcare Hamilton, Hamilton, Ontario L8P 3R2, Canada

2. Department of Psychiatry and Behavioural Neurosciences, McMaster University, Canada

3. Department of Psychiatry, UCSD, La Jolla, California, USA.

4. Division of Genetic Medicine, Vanderbilt University Medical Center, Nashville, Tennessee, USA.

5. Institute for Genomic Medicine, UCSD, La Jolla, CA, USA.

Table of Contents

[Phenotype and covariates description 2](#_Toc201313291)

[Delay discounting 2](#_Toc201313292)

[UPPS-P subscales 2](#_Toc201313293)

[Genetic ancestry calling 3](#_Toc201313294)

[Genetic data processing 4](#_Toc201313295)

[Autosome 4](#_Toc201313296)

[X chromosome 4](#_Toc201313297)

[Supplementary Figures 6](#_Toc201313298)

[References 15](#_Toc201313299)

# Phenotype and covariates description

## Delay discounting

Delay discounting was assessed using a computer-based adjusting-amount delay discounting procedure adapted for children (Luciana et al., 2018), following the method proposed by Koffarnus and Bickel (Koffarnus & Bickel, 2014). Participating children were presented with 42 hypothetical monetary choices, where they had to decide between receiving a smaller reward immediately or a larger reward ($100) after different delay intervals (6 hours, 1 day, 1 week, 1 month, 3 months, 1 year, and 5 years). This approach allowed for a detailed evaluation of delay discounting behavior. For more information, the user manual for the procedure utilized in the ABCD study is available here: <https://www.millisecond.com/download/library/v6/delaydiscountingtask/>.

The data have been filtered based on their goodness of fit to the hyperbolic regression model. Hyperbolic regression is a type of nonlinear regression used to model a hyperbolic relationship between two variables. In the context of decision making, it is commonly used to model delay discounting behavior, where an individual's valuation of a reward decreases as the delay to receiving it increases. The hyperbolic regression model can be represented by Equation 1, where V is the present value of the reward, A is the amount of the reward, D is the delay to receiving the reward, and k is a discounting parameter that determines the degree of discounting.

| $V=\frac{A}{1+KD}$ | 1 |
| --- | --- |

In Equation 1, as the delay D increases, the value V decreases asymptotically towards zero. The discounting parameter k determines the rate at which the value V decreases as a function of the delay D. A higher value of k indicates a steeper discounting curve, which means that the individual places a higher weight on immediate rewards compared to future rewards. A lower value of k indicates a flatter discounting curve, which means that the individual places a lower weight on immediate rewards compared to future rewards. The hyperbolic regression model can be fit to data using nonlinear regression techniques, such as least squares regression or maximum likelihood estimation. The resulting parameter estimates can be used to describe an individual's discounting behavior and can also be compared across groups or conditions to test hypotheses about the factors that influence delay discounting. The limited memory constrained Broyden–Fletcher–Goldfarb–Shanno (L-BFGS-B) optimization algorithm was used in R settings to achieve the optimized gradients for the model (Liu & Nocedal, 1989). The R^2^ score of the hyperbolic model was used to eliminate the nonsystematic readings of the delay discounting data. Ultimately, we chose a r-square filter of 0.5 to retain 50% of the variance explained to strike a balance between data quality and number of samples retained. . The continuous delay discounting phenotypes were first log10-transformed and then winsorised at the top 5% and 95% quantiles, replacing values above or below these quantiles, respectively.

## UPPS-P subscales

The ABCD Study administered a 20-item abbreviated youth version of the UPPS‑P Impulsive Behavior Scale (Luciana et al., 2018), specifically tailored for 9–10-year-olds. This version similarly includes five subscales, each composed of four items: negative urgency, positive urgency, sensation seeking, lack of premeditation (planning), and lack of perseverance. For each of these first order UPPS-P subscales, we computed raw subscale scores by summing the relevant item responses, in line with standard scoring procedures. No additional quality control steps (e.g., internal consistency filtering) were applied.

# Genetic ancestry calling

Genetic ancestry calling was done using the non-imputed ABCD V5.0 genetic data (ABCD_202209.updated.nodups.curated.cleaned_indivs). We first removed genetic variants in the high LD region generated (<https://genome.sph.umich.edu/wiki/Regions_of_high_linkage_disequilibrium_(LD)>) based on a previous report (Anderson et al., 2010). Then, we filtered SNPs based on independent pairwise LD (--indep-pairwise 150 50 0.5) in PLINK2. The same pre-processing was repeated on the 1000 Genomes data. After merging the ABCD samples and 1000 Genomes samples, 220,532 variants and 9,932 people passed SNP filters and sample QCs. We then generated the first two genetic principal components (PCs) after further QCs to retain autosomal SNPs with MAF > 0.05 and genotyping rate > 0.95.

The first two genetic PCs were used to visualize the samples and their ancestral origin (with only 1000 Genomes samples were annotated). A k-means clustering algorithm with 5 centers was used to call the genetic ancestry to be one of the continental population of “European”, “African”, “American”, “East Asian”, or “Other”.

|  | Cluster 1 | Cluster 2 | Cluster 3 | Cluster 4 | Cluster 5 |
| --- | --- | --- | --- | --- | --- |
| ABCD | 189 | 882 | 1488 | 62 | 5555 |
| AFR | 0 | 0 | 77 | 0 | 0 |
| AMR | 0 | 100 | 4 | 0 | 110 |
| EAS | 275 | 0 | 0 | 0 | 0 |
| EUR | 0 | 0 | 0 | 0 | 366 |

From the above table, we found high correspondence between cluster 5 and the “European” continental superpopulation of 1000 Genomes samples, and 1 and “East Asian”, and 2 and “American”, and 3 and “African”, and finally, 4 was not mapped to any of the four continental superpopulations. Thus, any ABCD samples mapped to 3 and 5 were assigned “African American” and “European American”, respectively.

We then merged these assigned clusters with the subset of participants passed full genotyping quality control, which removed an additional 7 participants with either self-identified sex not matching their genetically inferred sex or had failed to infer genetic sex. This left n=5,548 participants in the genetic European similarity group.

When compared with self-reported race, we observed that the correspondence between participants in the genetically European similarity group and self-reported White (4558/4574 = 99.7%) was high. While there was good agreement between the remaining genetic similarity groups and self-reported race, we did not include them in the analysis due to concerns for the low sample size once filtered by available impulsivity phenotypes. For example, the available sample size for participants in the African genetic similarity group would be n=483, which would not substantially contribute to the scientific rigor or statistical power of the study.

| Self-reported Ethnicity | Cluster 1 | Cluster 2 | Cluster 3 | Cluster 4 | Cluster 5 | Total |
| --- | --- | --- | --- | --- | --- | --- |
| White | 0 | 12 | 1 | 3 | 4558 | 4574 |
| Black | 0 | 0 | 1322 | 0 | 5 | 1327 |
| Hispanic | 2 | 763 | 44 | 3 | 686 | 1498 |
| Asian | 83 | 0 | 0 | 46 | 4 | 133 |
| Other | 19 | 19 | 116 | 9 | 295 | 458 |

# Genetic data processing

The SmokeScreen array was developed as a targeted genotyping array for addiction and substance use (Baurley et al., 2016). It covered the autosome, X chromosome (and pseudo-autosomal region), and the mitochondrial genome. We processed each of these set of genetic variants separately following the most up-to-date protocol and standards. For autosome, X chromosome, and mtDNA-CN, data QCs were done after genetic ancestry calling within each ancestry group. We also inferred haplogroup based on mtDNA genotype in the combined sample.

## Autosome

We first imputed genetic sex using the X chromosome genotypes from the Smokescreen binary PLINK Files. 203 samples with an *F* estimate between 0.2 and 0.8 were removed for problematic sex call, all remaining samples were assigned female (n_Female_ = 6,070; F < 0.2) or male (n_Male_ = 5,393; F>0.8). Following the notes on genetic data from ABCD (<https://wiki.abcdstudy.org/release-notes/non-imaging/genetics.html>), we retained unrelated individuals based on the estimated kinship matrix derived using GENESIS (Gogarten et al., 2019)provided as part of the data release (“ABCD_202209.updated.nodups.curated_unrelateds.txt”).

## X chromosome

For Xchr variants, we further filtered based on a minimal allele count of 30 to ensure that the higher degrees of freedom model produced a good fit to the data. These resulted in 521,964 and 389,048 variants for association testing in AA and EA samples.

# Supplementary Figures

**Figure S1. Scatterplots of the first two genetic principal components based on the combined ABCD samples and cosmopolitan samples from the 1000 Genomes Project.**

Panel A shows a scatterplot of the first two principal components (PCs) for the combined study samples and 1000 Genomes Project samples, with different colors to indicate the subcontinental populations of origin. Panel B shows a scatterplot of the first two PCs for the combined samples, with different colors to indicate the continental origins of the samples. The study samples are shown in gray dots and only those overlapped with the European/African subset of the 1000 Genome Project samples were retained for the analysis.
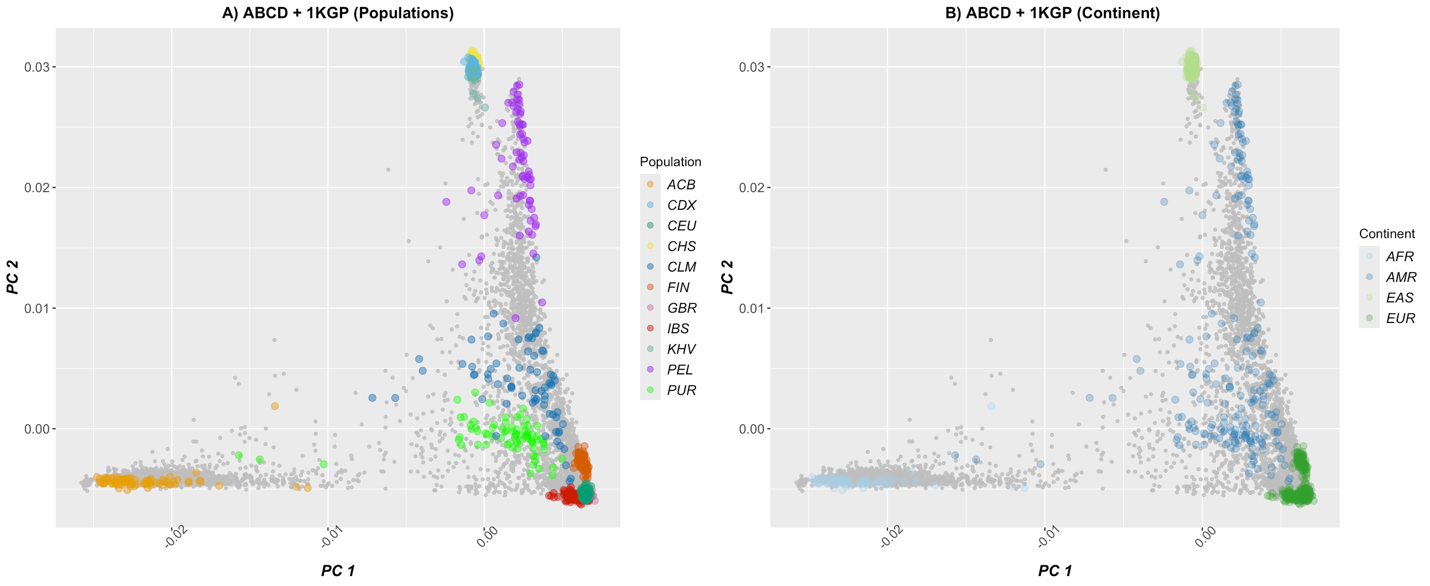


**Figure S2. Consort diagram of genetic variant and sample quality controls in ABCD.**

A consort diagram that shows the inclusion and exclusion of sample and SNP data at each data processing stage.

**Figure S3. Histogram of impulsivity phenotypes stratified by sex.**

The histograms illustrate the distribution of impulsivity measures stratified by sex. The x-axis represents values of each impulsivity measure, range from 4-20 for UPPS-P subscales and -3.1 to -0.22 for delay discounting.

**Figure S4. Genetic and phenotypic correlation among impulsivity phenotypes in three studies.**


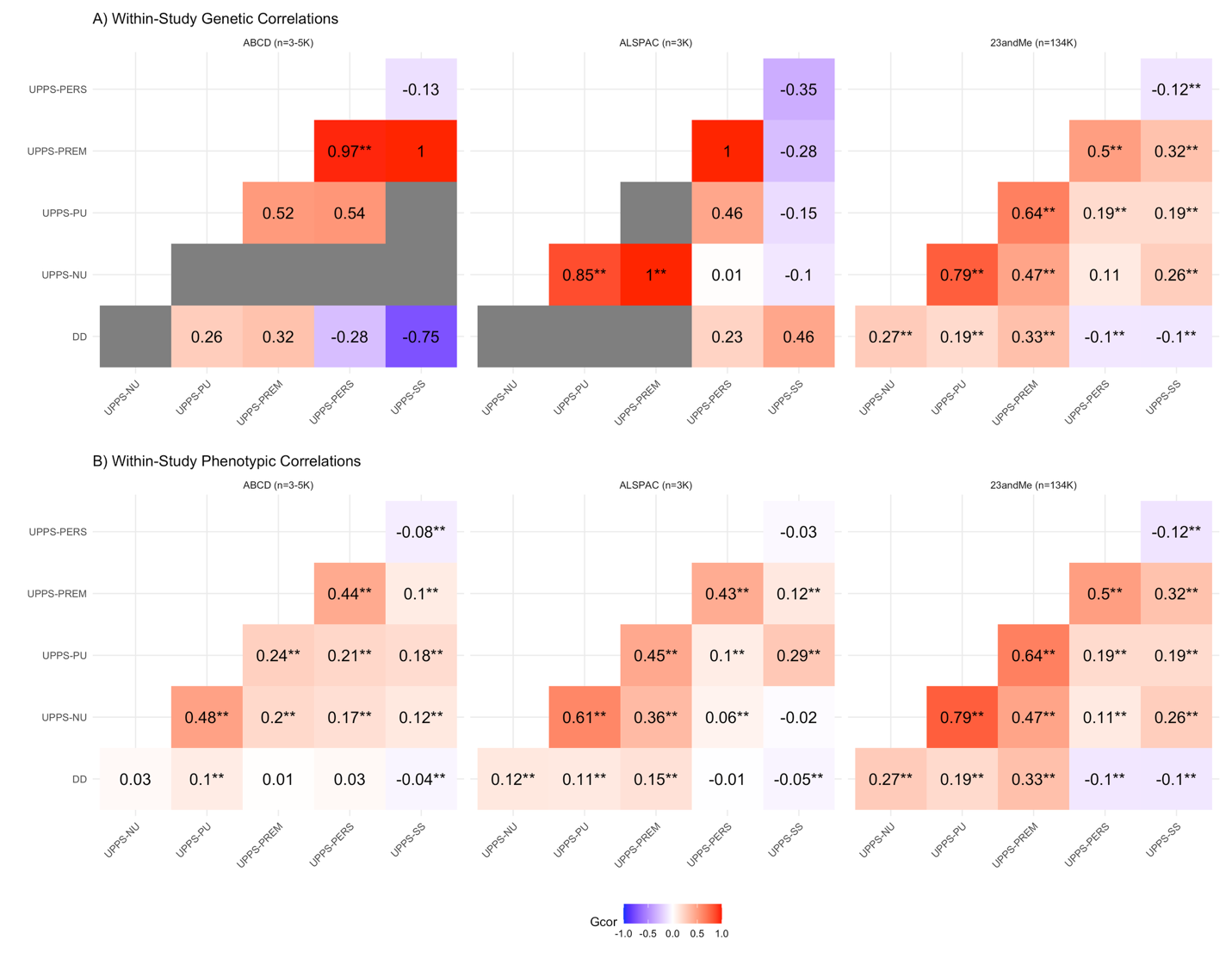
This figure presents within-study correlations among delay discounting and UPPS-P impulsivity subscales across three cohorts: ABCD (n = 3–5K), ALSPAC (n = 3K), and 23andMe (n = 134K). Panel A) displays SNP-based genetic correlations (r_g_), while Panel B) shows phenotypic correlations (rₚ), with each matrix color-coded by the direction and magnitude of the correlation (positive in red, negative in blue). Gray tiles indicate correlations that were not estimable. Asterisks denote statistical significance (*p* < 0.05).

**Figure S5. Genetic correlation of impulsivity phenotypes across three studies.** This figure presents between-study correlations among delay discounting and UPPS-P impulsivity subscales for three cohorts: ABCD (n = 3–5K), ALSPAC (n = 3K), and 23andMe (n = 134K). Each matrix is color-coded by the magnitude of the correlation (positive in red, null in white). Gray tiles indicate correlations that were not estimable. Asterisks denote statistical significance (*p* < 0.05).


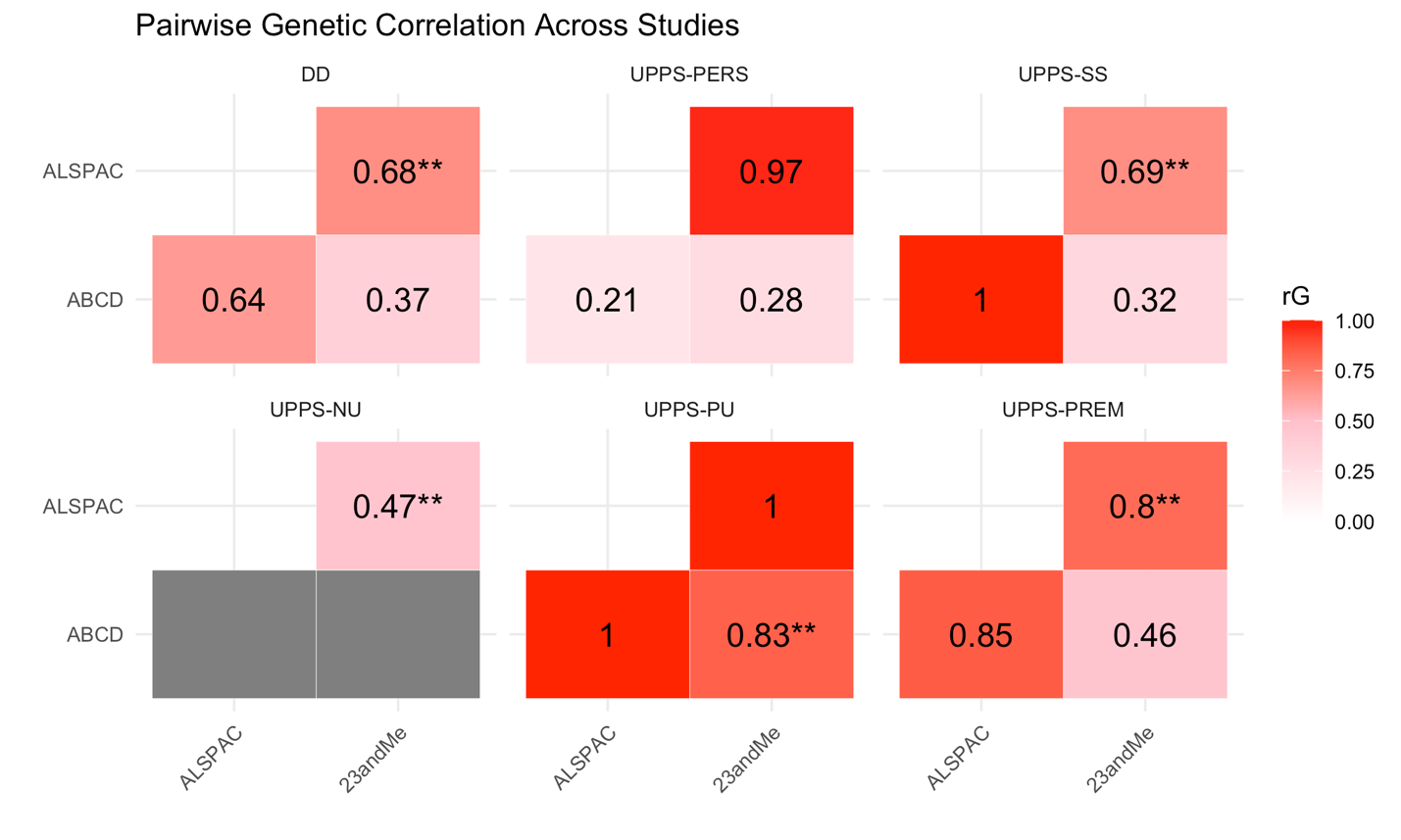


**Figure S6. Manhattan plot of genome-wide association results for the impulsivity phenotypes.** Manhattan plots for the 6 impulsivity phenotypes were merged in the same figure. The y-axis shows the -log10(p-value), with a red horizontal line indicating the genome-wide significance threshold. The colored dots represent SNPs reaching marginal significance (*p* < 1×10^-5^) for each respective impulsivity phenotype.


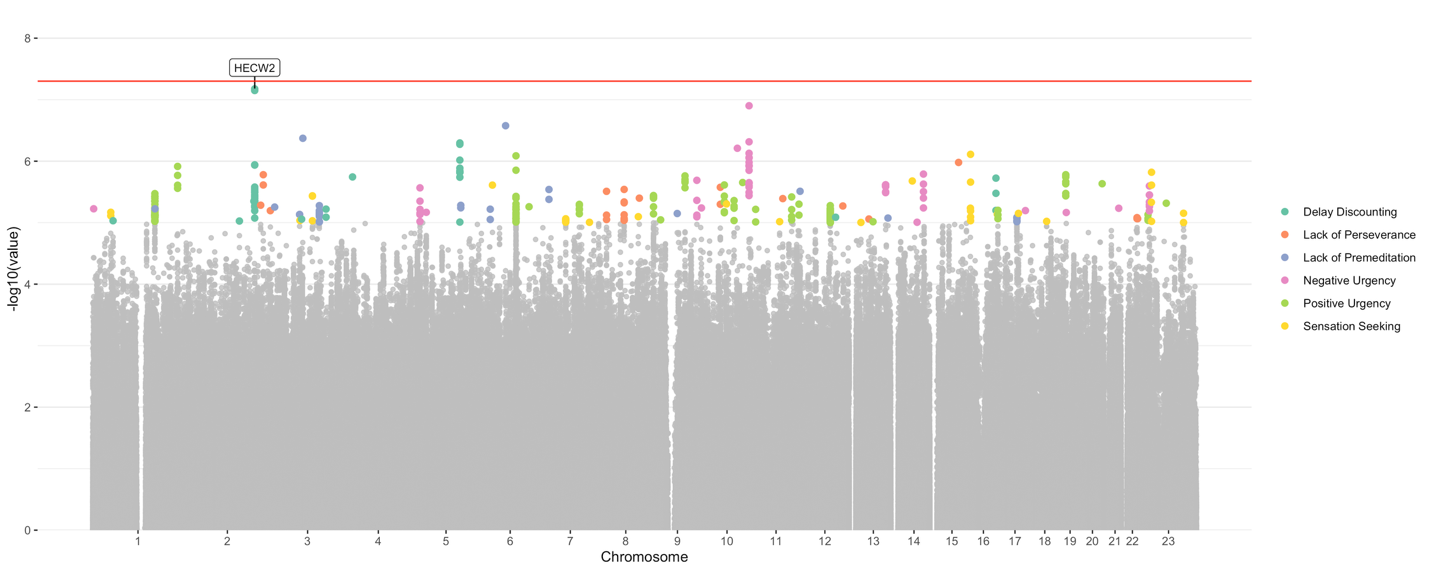


**Figure S7. Quantile-quantile plots of genome-wide *p*-values of autosomal SNPs for impulsivity phenotypes.**

Quantile-quantile plots summarized the genome-wide *p*-values between each SNP and impulsivity phenotype for autosomal SNPs. The red line (y=x) is the line of reference and the genomic inflation factor, calculated as the ratio between the observed median and the theoretical median of the association test statistics, was annotated for each outcome.


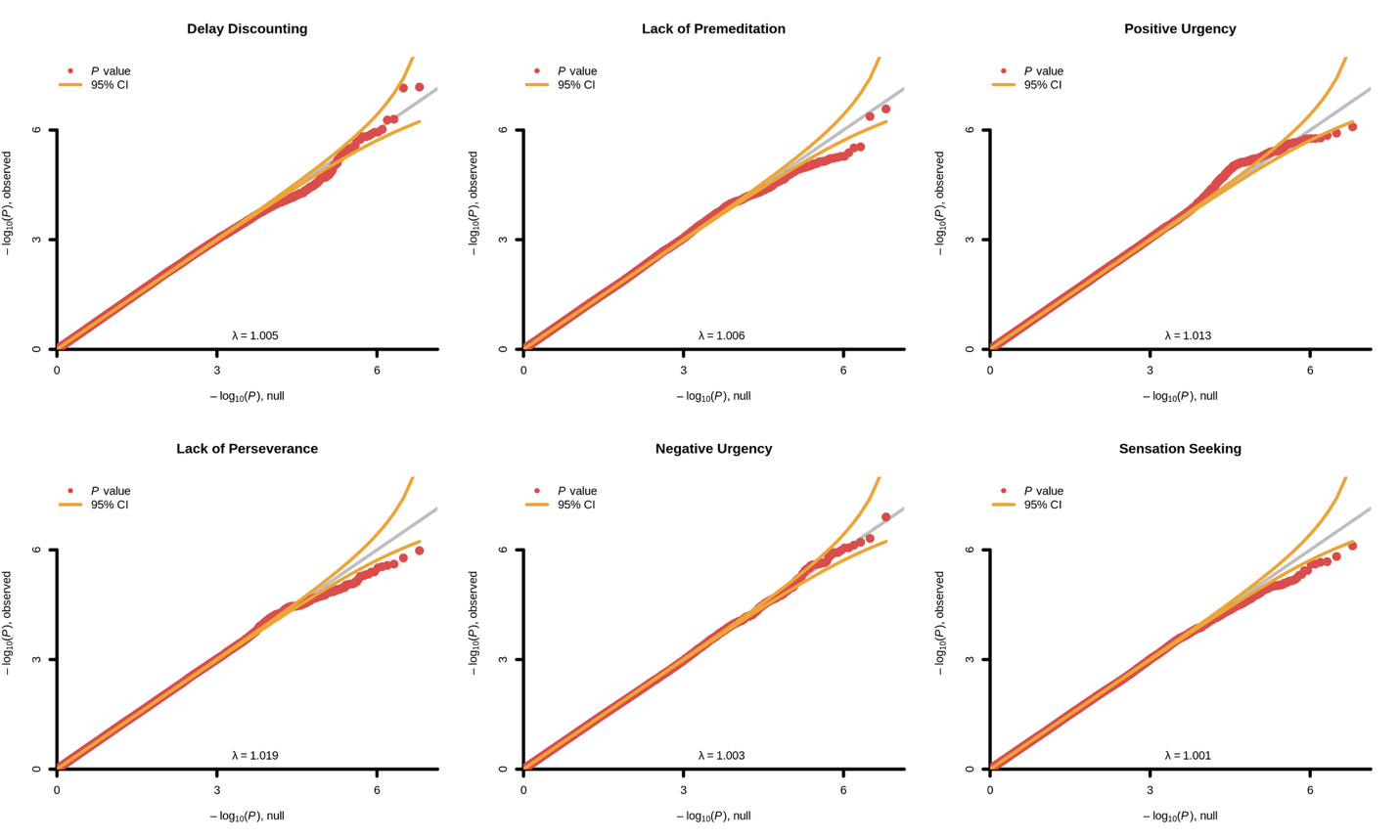


**Figure S8. Locus Zoom plot for DD at the *HECW2* locus.**

This locus zoom plot shows the association between genetic variants in the *HECW2* gene locus and delay discounting. The x-axis represents the chromosomal position, while the y-axis displays the –log10(p-value) of the association for each variant. The lead SNP (rs3820908) is marked with a purple diamond, and the color gradient of surrounding points indicates the degree of linkage disequilibrium (LD) with the lead SNP, ranging from red (high LD) to blue (low LD) in all populations. Gene annotations are provided below the plot to highlight relevant genes in the region, and recombination rates are shown in the background, indicating areas of higher genetic recombination. The vertical lines under GWAS catalog annotation show the overlap of SNPs in this region that had previously been reported at genome-wide significance.


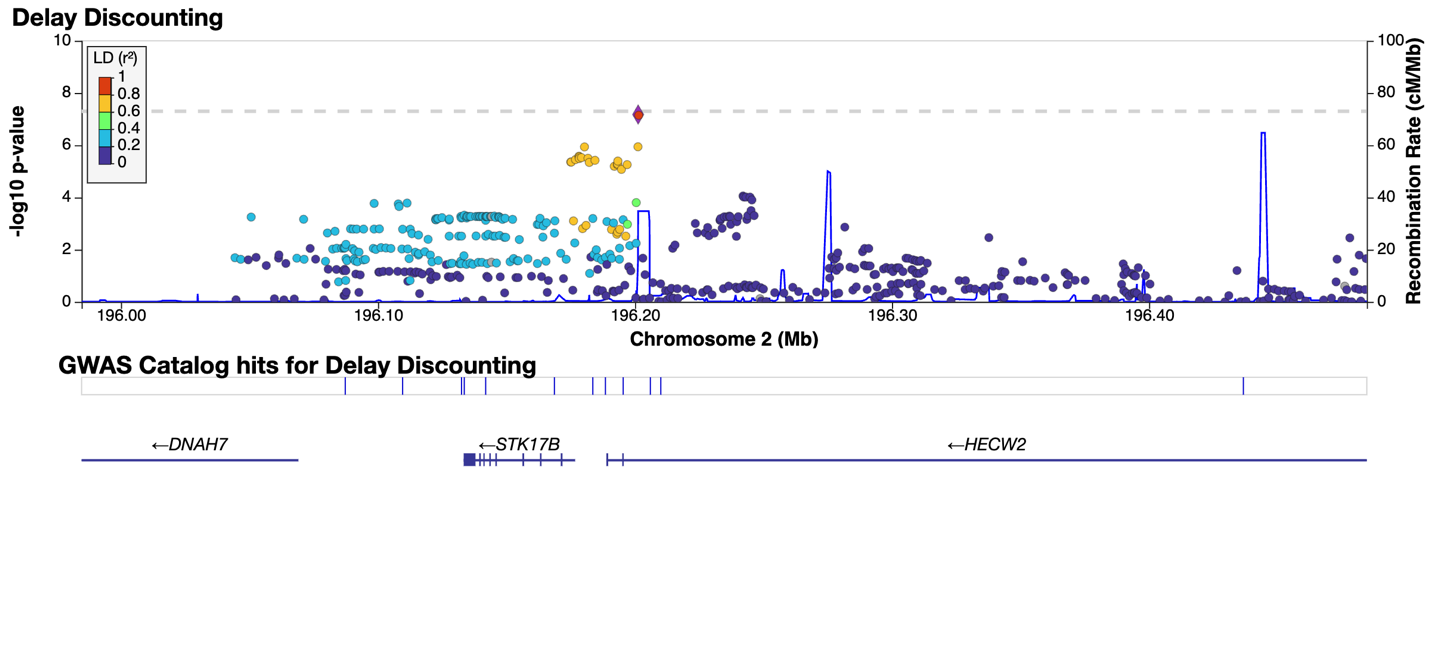


**Figure S9. Comparing the contribution of PGSs to impulsivity trait variance.**

This figure displays the adjusted R² values and corresponding 95% bootstrap confidence intervals for polygenic score (PGS) prediction models across six impulsivity traits: Delay Discounting, Lack of Perseverance, Lack of Planning, Negative Urgency, Positive Urgency, and Sensation Seeking. Four PGS models are compared: (1) trait-specific PGS alone (black), (2) all available adult-derived PGSs (orange), (3) all PGSs excluding the trait-specific PGS (blue), and (4) a model comparing the variance explained by trait PGS and externalizing PGS vs. externalizing PGS (red). Across traits, models incorporating multiple PGSs generally explained more variance than trait PGS alone. Asterisks above each point indicate significance based on bootstrap p-values, with *p* < 0.05 (*), *p* < 0.01 (**), and *p* < 0.001 (***).


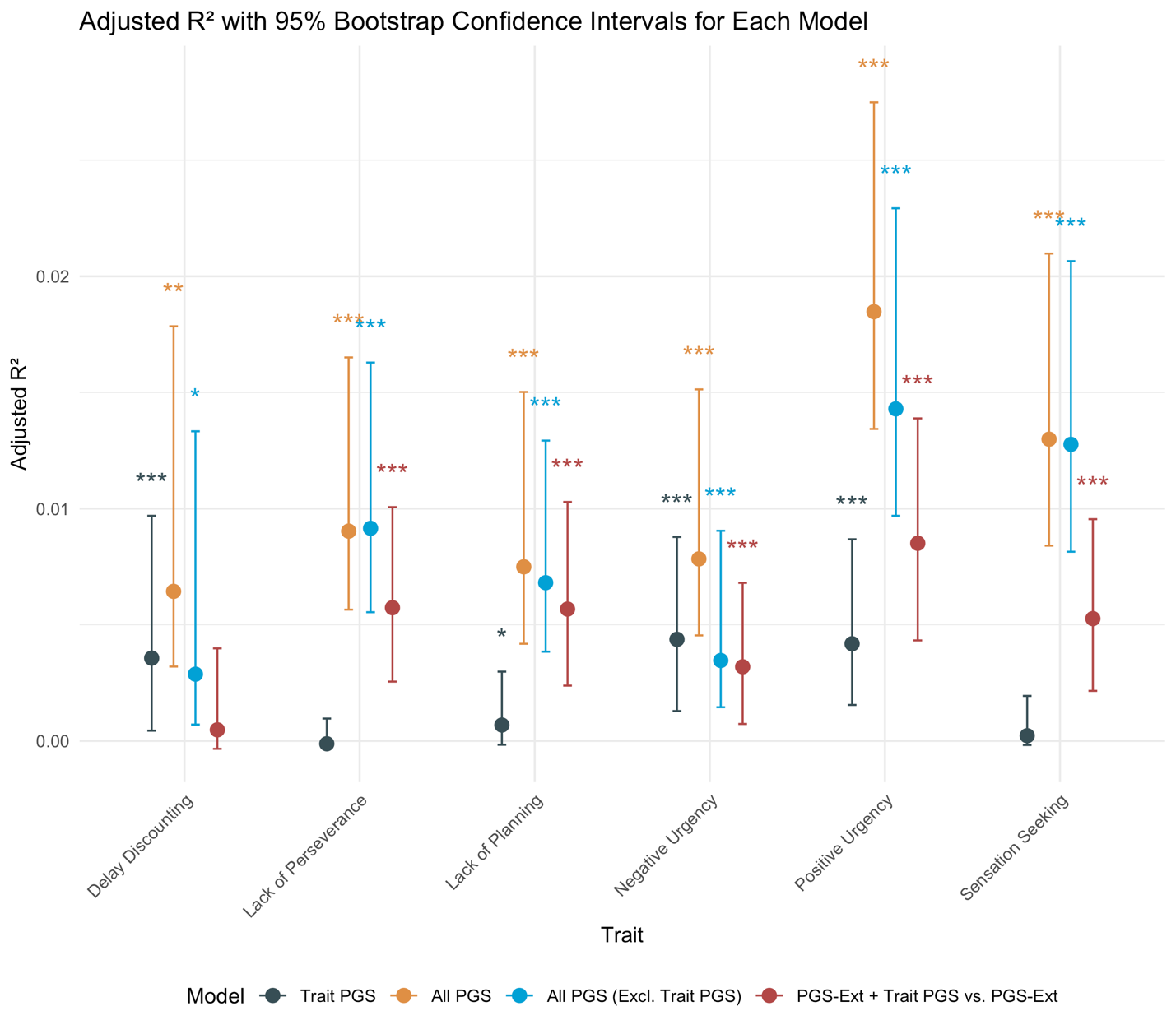


# References

Anderson, C. A., Pettersson, F. H., Clarke, G. M., Cardon, L. R., Morris, A. P., & Zondervan, K. T. (2010). Data quality control in genetic case-control association studies. *Nature Protocols*, *5*(9). https://doi.org/10.1038/nprot.2010.116

Baurley, J. W., Edlund, C. K., Pardamean, C. I., Conti, D. V., & Bergen, A. W. (2016). Smokescreen: A targeted genotyping array for addiction research. *BMC Genomics*, *17*(1). https://doi.org/10.1186/s12864-016-2495-7

Gogarten, S. M., Sofer, T., Chen, H., Yu, C., Brody, J. A., Thornton, T. A., Rice, K. M., & Conomos, M. P. (2019). Genetic association testing using the GENESIS R/Bioconductor package. *Bioinformatics (Oxford, England)*, *35*(24), 5346–5348. https://doi.org/10.1093/BIOINFORMATICS/BTZ567

Koffarnus, M. N., & Bickel, W. K. (2014). A 5-trial adjusting delay discounting task: Accurate discount rates in less than one minute. *Experimental and Clinical Psychopharmacology*, *22*(3). https://doi.org/10.1037/a0035973

Liu, D. C., & Nocedal, J. (1989). On the limited memory BFGS method for large scale optimization. *Mathematical Programming*, *45*(1–3), 503–528. https://doi.org/10.1007/BF01589116/METRICS

Luciana, M., Bjork, J. M., Nagel, B. J., Barch, D. M., Gonzalez, R., Nixon, S. J., & Banich, M. T. (2018). Adolescent neurocognitive development and impacts of substance use: Overview of the adolescent brain cognitive development (ABCD) baseline neurocognition battery. *Developmental Cognitive Neuroscience*, *32*, 67–79. https://doi.org/10.1016/J.DCN.2018.02.006
