# Supplementary material for: Genome‐Wide Association Studies of Delay Discounting and Impulsive Personality Traits in Children From the Adolescent Behavior and Cognitive Development Study
Source: Genes Brain Behav. 2025 Aug 23;24(4):e70033. doi: 10.1111/gbb.70033 (PMC12374252; doi:10.1111/gbb.70033)
Supplement: Supplementary file 1 — Data S1: Supporting Information. [file GBB-24-e70033-s001.zip › gbb70033-sup-0004-Supinfo1@Suppl-Materials-Genomics_of_impulsivity_ABCD.docx]

**Supplementary Materials for “**Genome-wide association studies of delay discounting and impulsive personality traits in children from the Adolescent Behavior and Cognitive Development Study”

Wei Q. Deng1,2,*, Mahmoud Elsayed1,2, Kyla L. Belisario1,2, Sandra Sanchez-Roige3,4,5, Abraham A. Palmer3,5, James MacKillop1,2*

1. Peter Boris Centre for Addictions Research, St. Joseph’s Healthcare Hamilton, Hamilton, Ontario L8P 3R2, Canada

2. Department of Psychiatry and Behavioural Neurosciences, McMaster University, Canada

3. Department of Psychiatry, UCSD, La Jolla, California, USA.

4. Division of Genetic Medicine, Vanderbilt University Medical Center, Nashville, Tennessee, USA.

5. Institute for Genomic Medicine, UCSD, La Jolla, CA, USA.

Table of Contents

[Phenotype and covariates description 2](#_Toc177395258)

[Delay discounting 2](#_Toc177395259)

[UPPS-P factors 2](#_Toc177395260)

[Genetic ancestry calling 3](#_Toc177395261)

[Genetic data processing 4](#_Toc177395262)

[Autosome 4](#_Toc177395263)

[X chromosome 4](#_Toc177395264)

[Mitochondrial haplogroup and genotype 4](#_Toc177395265)

[References 5](#_Toc177395266)

# Phenotype and covariates description

## Delay discounting

Delay discounting was assessed using a computer-based adjusting-amount delay discounting procedure adapted for children (Luciana et al., 2018), following the method proposed by Koffarnus and Bickel (Koffarnus & Bickel, 2014). Participating children were presented with 42 hypothetical monetary choices, where they had to decide between receiving a smaller reward immediately or a larger reward ($100) after different delay intervals (6 hours, 1 day, 1 week, 1 month, 3 months, 1 year, and 5 years). This approach allowed for a detailed evaluation of delay discounting behavior. For more information, the user manual for the procedure utilized in the ABCD study is available here: <https://www.millisecond.com/download/library/v6/delaydiscountingtask/>.

The data have been filtered based on their goodness of fit to the hyperbolic regression model. Hyperbolic regression is a type of nonlinear regression used to model a hyperbolic relationship between two variables. In the context of decision making, it is commonly used to model delay discounting behavior, where an individual's valuation of a reward decreases as the delay to receiving it increases. The hyperbolic regression model can be represented by Equation 1, where V is the present value of the reward, A is the amount of the reward, D is the delay to receiving the reward, and k is a discounting parameter that determines the degree of discounting.

| $V=\frac{A}{1+KD}$ | 1 |
| --- | --- |

In Equation 1, as the delay D increases, the value V decreases asymptotically towards zero. The discounting parameter k determines the rate at which the value V decreases as a function of the delay D. A higher value of k indicates a steeper discounting curve, which means that the individual places a higher weight on immediate rewards compared to future rewards. A lower value of k indicates a flatter discounting curve, which means that the individual places a lower weight on immediate rewards compared to future rewards. The hyperbolic regression model can be fit to data using nonlinear regression techniques, such as least squares regression or maximum likelihood estimation. The resulting parameter estimates can be used to describe an individual's discounting behavior and can also be compared across groups or conditions to test hypotheses about the factors that influence delay discounting. The limited memory constrained Broyden–Fletcher–Goldfarb–Shanno (L-BFGS-B) optimization algorithm was used in R settings to achieve the optimized gradients for the model (Liu & Nocedal, 1989). The R^2^ score of the hyperbolic model was used to eliminate the nonsystematic readings of the delay discounting data. Ultimately, we chose a r-square filter of 0.5 to retain 50% of the variance explained to strike a balance between data quality and number of samples retained. . The continuous delay discounting phenotypes were first log10-transformed and then winsorised at the top 5% and 95% quantiles, replacing values above or below these quantiles, respectively.

## UPPS-P subscales

For each of the first order UPPS-P subscales, we summed up the respective items without further quality control steps to calculate negative urgency, positive urgency, sensation seeking, lack of planning, and lack of perseverance, respectively.

# Genetic ancestry calling

Genetic ancestry calling was done using the non-imputed ABCD V5.0 genetic data (ABCD_202209.updated.nodups.curated.cleaned_indivs). We first removed genetic variants in the high LD region generated (<https://genome.sph.umich.edu/wiki/Regions_of_high_linkage_disequilibrium_(LD)>) based on a previous report (Anderson et al., 2010). Then, we filtered SNPs based on independent pairwise LD (--indep-pairwise 150 50 0.5) in PLINK2. The same pre-processing was repeated on the 1000 Genomes data. After merging the ABCD samples and 1000 Genomes samples, 220,532 variants and 9,932 people passed SNP filters and sample QCs. We then generated the first two genetic principal components (PCs) after further QCs to retain autosomal SNPs with MAF > 0.05 and genotyping rate > 0.95.

The first two genetic PCs were used to visualize the samples and their ancestral origin (with only 1000 Genomes samples were annotated). A k-means clustering algorithm with 5 centers was used to call the genetic ancestry to be one of the continental population of “European”, “African”, “American”, “East Asian”, or “Other”.

|  | Cluster 1 | Cluster 2 | Cluster 3 | Cluster 4 | Cluster 5 |
| --- | --- | --- | --- | --- | --- |
| ABCD | 403 | 941 | 1773 | 164 | 5719 |
| AFR | 0 | 0 | 77 | 0 | 0 |
| AMR | 0 | 100 | 4 | 0 | 110 |
| EAS | 275 | 0 | 0 | 0 | 0 |
| EUR | 0 | 0 | 0 | 0 | 366 |

From the above table, we found high correspondence between cluster 5 and the “European” continental superpopulation of 1000 Genomes samples, and 1 and “East Asian”, and 2 and “American”, and 3 and “African”, and finally, 4 was not mapped to any of the four continental superpopulations. Thus, any ABCD samples mapped to 3 and 5 were assigned “African American” and “European American”, respectively.

When compared with self-reported ethnicity, we observed that the correspondence between genetically Europeans and self-reported White (4558/4574 = 99.7%), and between genetically African and self-reported Black (1322/1327 = 99.6%) was high.

| Self-reported Ethnicity | Cluster 1 | Cluster 2 | Cluster 3 | Cluster 4 | Cluster 5 | Total |
| --- | --- | --- | --- | --- | --- | --- |
| White | 0 | 12 | 1 | 3 | 4558 | 4574 |
| Black | 0 | 0 | 1322 | 0 | 5 | 1327 |
| Hispanic | 2 | 763 | 44 | 3 | 686 | 1498 |
| Asian | 83 | 0 | 0 | 46 | 4 | 133 |
| Other | 19 | 19 | 116 | 9 | 295 | 458 |

# Genetic data processing

The SmokeScreen array was developed as a targeted genotyping array for addiction and substance use (Baurley et al., 2016). It covered the autosome, X chromosome (and pseudo-autosomal region), and the mitochondrial genome. We processed each of these set of genetic variants separately following the most up-to-date protocol and standards. For autosome, X chromosome, and mtDNA-CN, data QCs were done after genetic ancestry calling within each ancestry group. We also inferred haplogroup based on mtDNA genotype in the combined sample.

## Autosome

We first imputed genetic sex using the X chromosome genotypes from the Smokescreen binary PLINK Files. 203 samples with an *F* estimate between 0.2 and 0.8 were removed for problematic sex call, all remaining samples were assigned female (n_Female_ = 6,070; F < 0.2) or male (n_Male_ = 5,393; F>0.8). Following the notes on genetic data from ABCD (<https://wiki.abcdstudy.org/release-notes/non-imaging/genetics.html>), we retained unrelated individuals based on the estimated kinship matrix derived using GENESIS (Gogarten et al., 2019)provided as part of the data release (“ABCD_202209.updated.nodups.curated_unrelateds.txt”).

## X chromosome

For Xchr variants, we further filtered based on a minimal allele count of 30 to ensure that the higher degrees of freedom model produced a good fit to the data. These resulted in 521,964 and 389,048 variants for association testing in AA and EA samples.

## Mitochondrial haplogroup and genotype

The mtDNA data were genotyped on the smokescreen array with a total of 180 variants measured. We first confirmed the mitochondrial DNA variant alleles and their nucleotide positions conform to the revised Cambridge Reference Sequence (rCRS). To be consistent across studies, the marker names were created as ``MT-’’ and position numbers under the rCRS. The genotyping rate was high (> 0.9) and missing rate low (< 0.1). We then proceeded with additional mitochondrial-specific procedures. The mtDNA alleles were compared with the reference alleles defined in the rCRS sequence <https://www.mitomap.org/MITOMAP/HumanMitoSeq>. Those with mismatched alleles (n=6) and monomorphic (n=10) were removed, leaving 164 mtDNA SNPs for subsequent analysis. After filtering, 54 and 94 common variants (MAF > 0.01) remained in AA and EA samples, respectively.

***Haplogroup Classification and Association Analysis*** Following the lineage-based approach to mtDNA analyses, we attempted to classify samples to haplogroups that represent related groups that share maternal ancestry. Haplogroup classification was performed using Haplogrep (Weissensteiner et al., 2016) on variants with MAF > 0.001. The haplogroup assignments were done in n=11,100 samples, with the majority achieving a quality score > 0.9 (n=11,012). For subsequent analysis, we focused on the 6,795 samples in the EA and AA subsets after genetic and phenotype QC. We also removed haplogroups with <20 samples (E, F, Z). The distribution of the haplogroups agrees with that of EA and AA ancestries in this subsample (Suppl. Table 11).

***mtSNP Association Analysis*** We analyzed common variants (MAF > 0.01) for association with impulsivity phenotypes in AA and EA samples separately. For common variant associations, PLINK was used to analyze the genotype data through a similar linear regression model, adjusted for genetic sex, age, parental income, and the first 20 autosomal PC covariates.

# References

Anderson, C. A., Pettersson, F. H., Clarke, G. M., Cardon, L. R., Morris, A. P., & Zondervan, K. T. (2010). Data quality control in genetic case-control association studies. *Nature Protocols*, *5*(9). https://doi.org/10.1038/nprot.2010.116

Baurley, J. W., Edlund, C. K., Pardamean, C. I., Conti, D. V., & Bergen, A. W. (2016). Smokescreen: A targeted genotyping array for addiction research. *BMC Genomics*, *17*(1). https://doi.org/10.1186/s12864-016-2495-7

Gogarten, S. M., Sofer, T., Chen, H., Yu, C., Brody, J. A., Thornton, T. A., Rice, K. M., & Conomos, M. P. (2019). Genetic association testing using the GENESIS R/Bioconductor package. *Bioinformatics (Oxford, England)*, *35*(24), 5346–5348. https://doi.org/10.1093/BIOINFORMATICS/BTZ567

Koffarnus, M. N., & Bickel, W. K. (2014). A 5-trial adjusting delay discounting task: Accurate discount rates in less than one minute. *Experimental and Clinical Psychopharmacology*, *22*(3). https://doi.org/10.1037/a0035973

Liu, D. C., & Nocedal, J. (1989). On the limited memory BFGS method for large scale optimization. *Mathematical Programming*, *45*(1–3), 503–528. https://doi.org/10.1007/BF01589116/METRICS

Luciana, M., Bjork, J. M., Nagel, B. J., Barch, D. M., Gonzalez, R., Nixon, S. J., & Banich, M. T. (2018). Adolescent neurocognitive development and impacts of substance use: Overview of the adolescent brain cognitive development (ABCD) baseline neurocognition battery. *Developmental Cognitive Neuroscience*, *32*, 67–79. https://doi.org/10.1016/J.DCN.2018.02.006

Weissensteiner, H., Pacher, D., Kloss-Brandstätter, A., Forer, L., Specht, G., Bandelt, H. J., Kronenberg, F., Salas, A., & Schönherr, S. (2016). HaploGrep 2: mitochondrial haplogroup classification in the era of high-throughput sequencing. *Nucleic Acids Research*, *44*(W1). https://doi.org/10.1093/nar/gkw233
