# Supplementary figures and images for: Genome‐Wide Association Studies of Delay Discounting and Impulsive Personality Traits in Children From the Adolescent Behavior and Cognitive Development Study
Source: Genes Brain Behav. 2025 Aug 23;24(4):e70033. doi: 10.1111/gbb.70033 (PMC12374252; doi:10.1111/gbb.70033)

# Histograms of Impulsivity by Sex

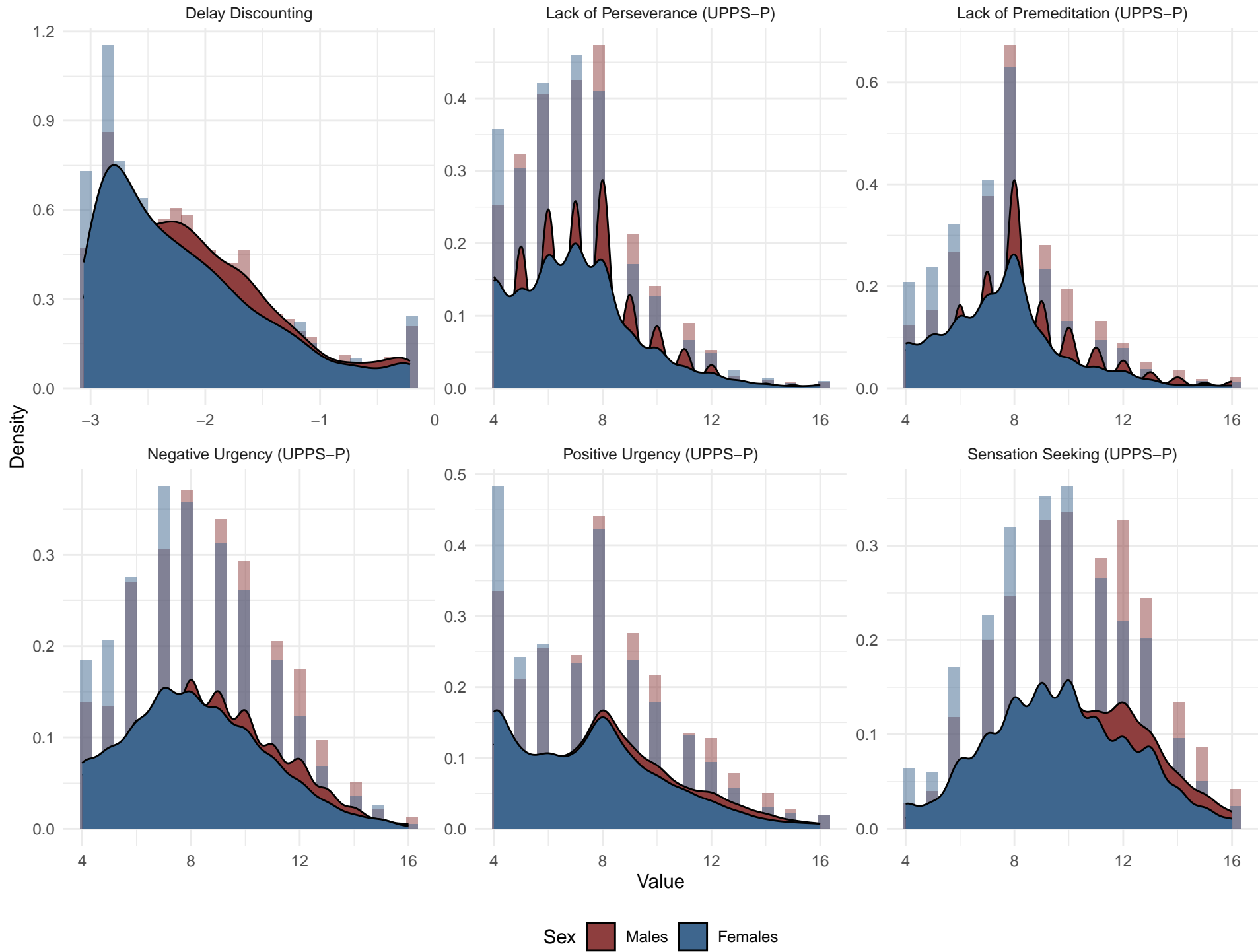

Supplement: Supplementary file 1 — Data S1: Supporting Information. [file GBB-24-e70033-s001.zip › GBB_70033_f3_SF3-ABCD_hist_impulsivity.pdf]

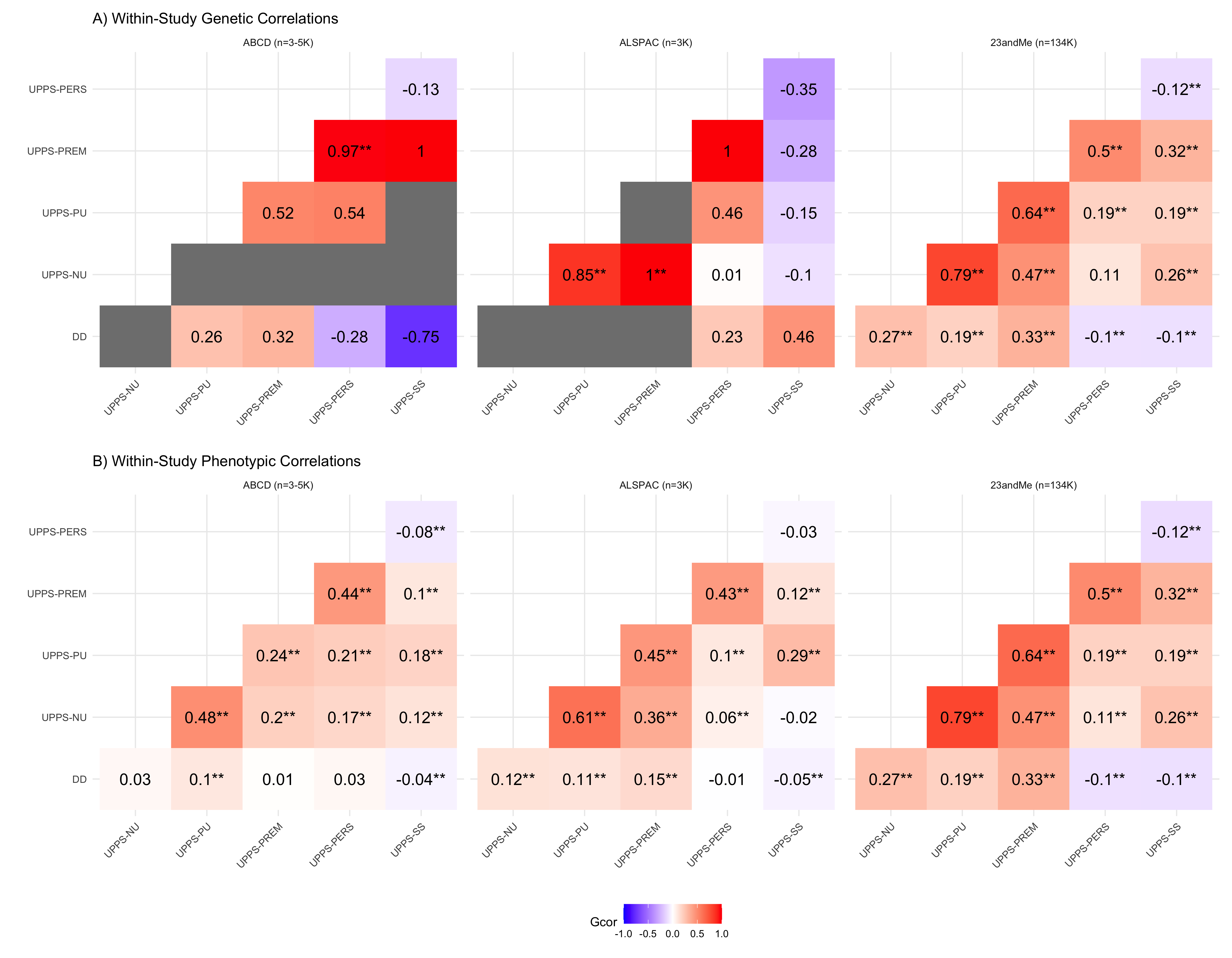

Supplement: Supplementary file 1 — Data S1: Supporting Information. [file GBB-24-e70033-s001.zip › GBB_70033_f4_SF4-co-h2_within_study.png]

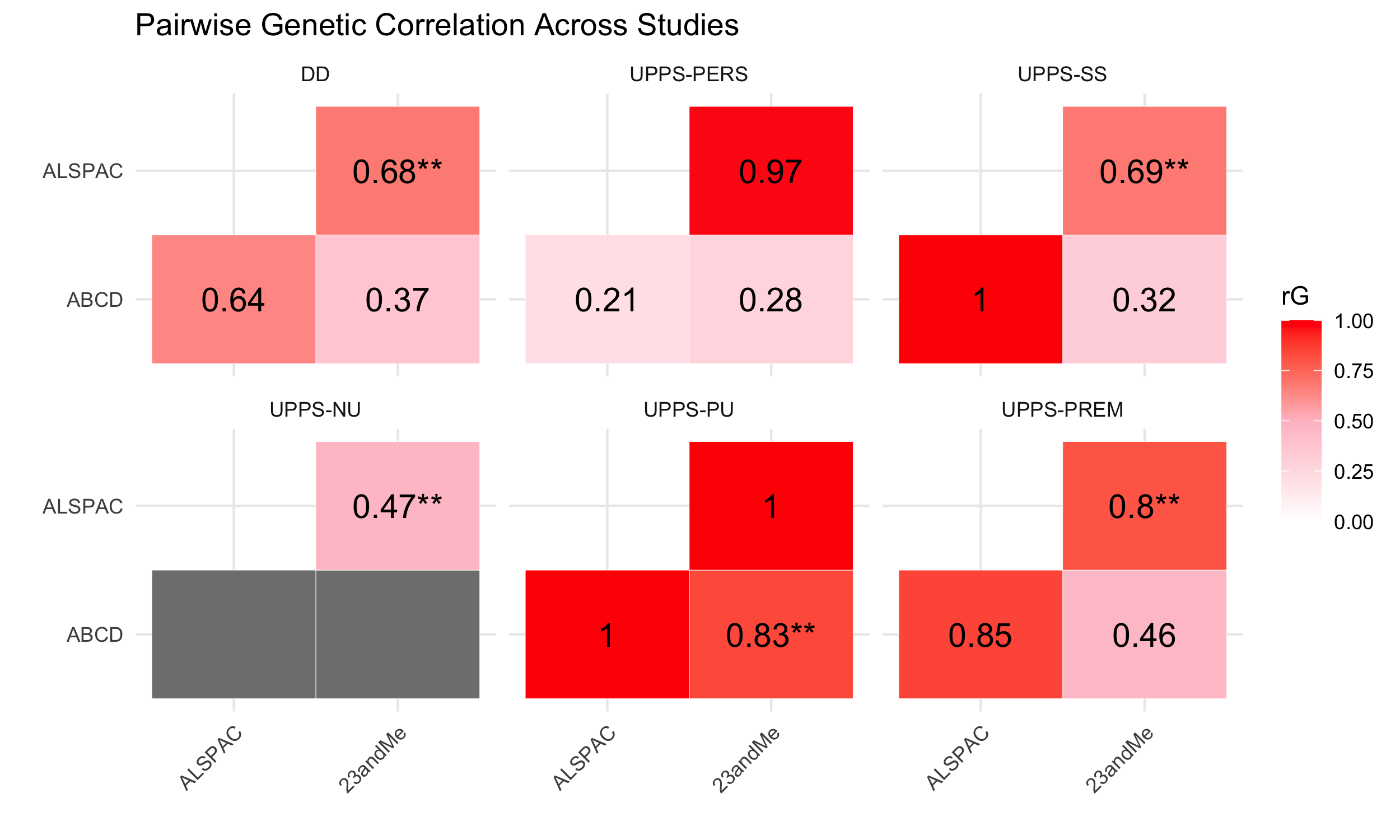

Supplement: Supplementary file 1 — Data S1: Supporting Information. [file GBB-24-e70033-s001.zip › GBB_70033_f5_SF5-GCOR-imp.png]

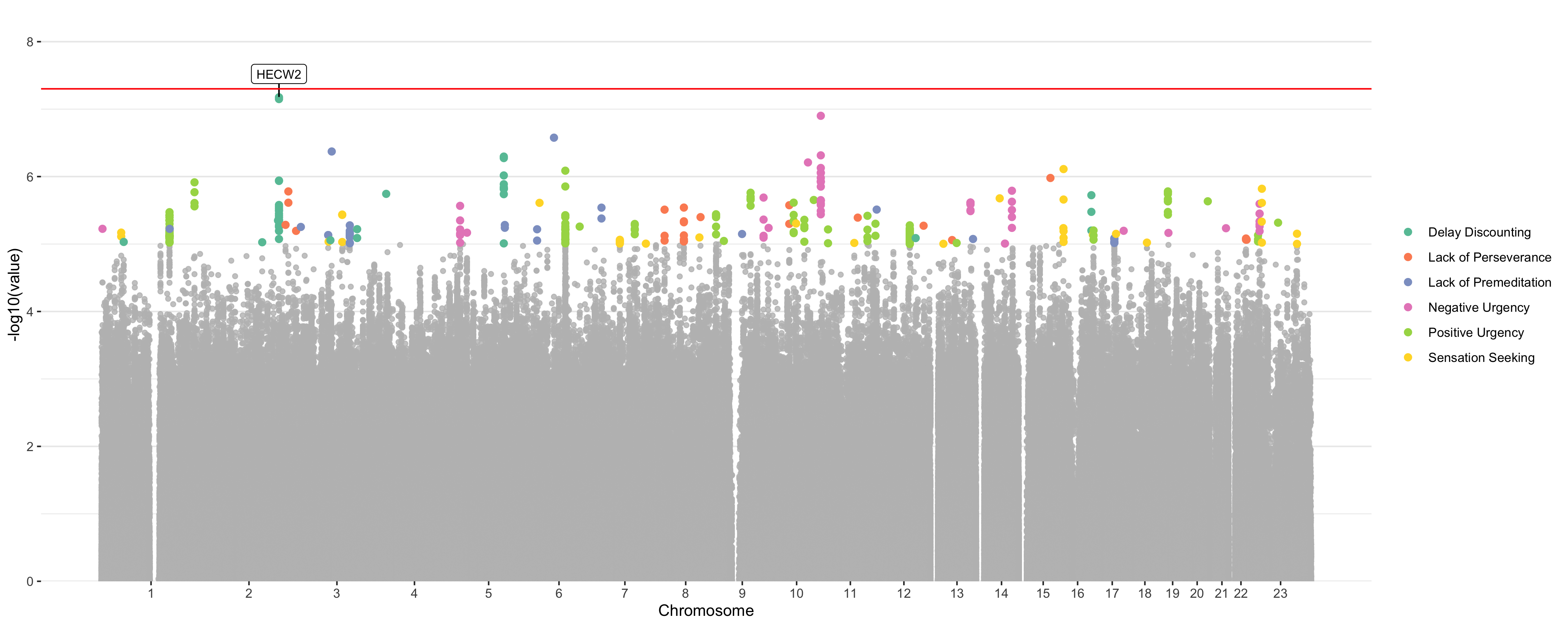

Supplement: Supplementary file 1 — Data S1: Supporting Information. [file GBB-24-e70033-s001.zip › GBB_70033_f6_SF6-MAN.png]

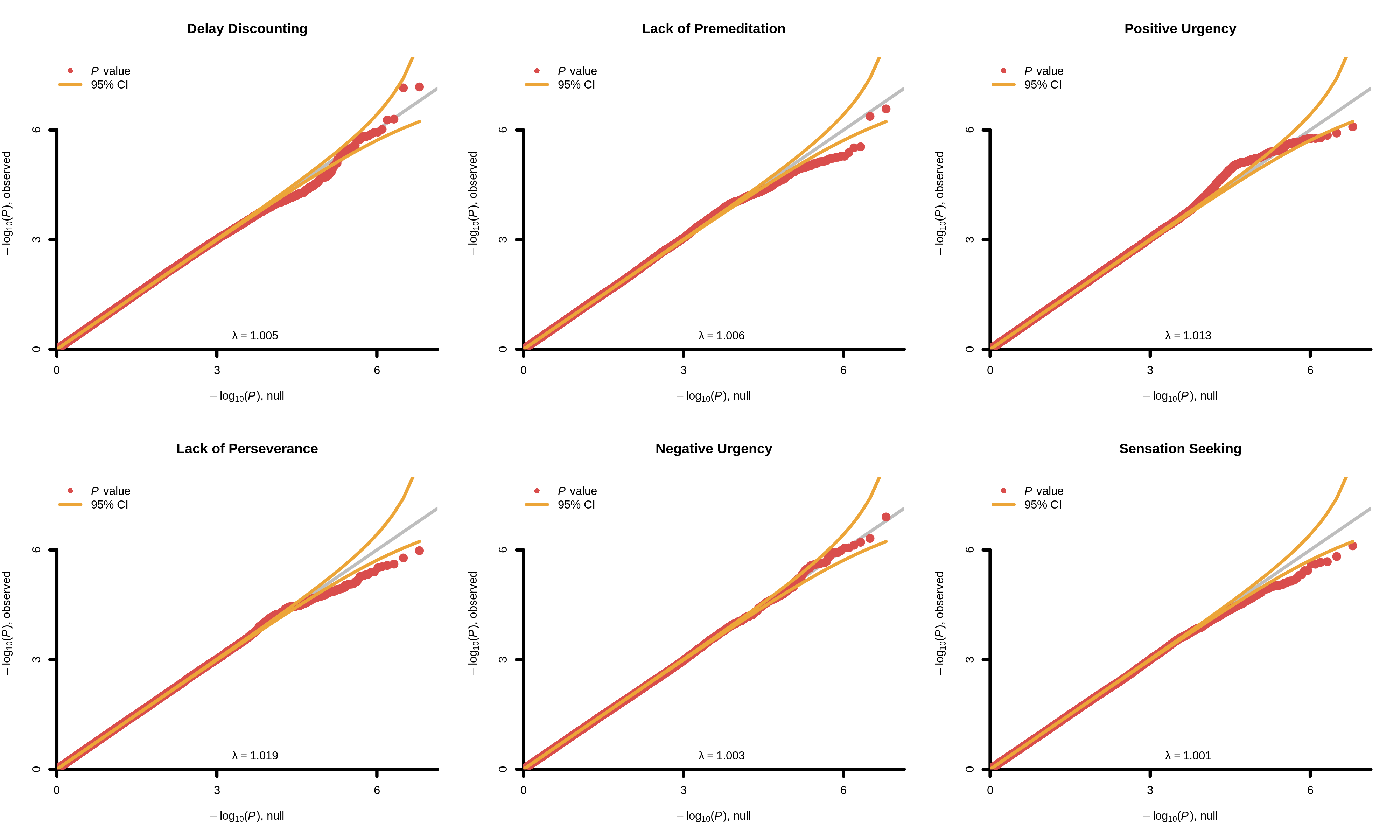

Supplement: Supplementary file 1 — Data S1: Supporting Information. [file GBB-24-e70033-s001.zip › GBB_70033_f7_SF7-QQ-plot-EA-all_updated2025.png]

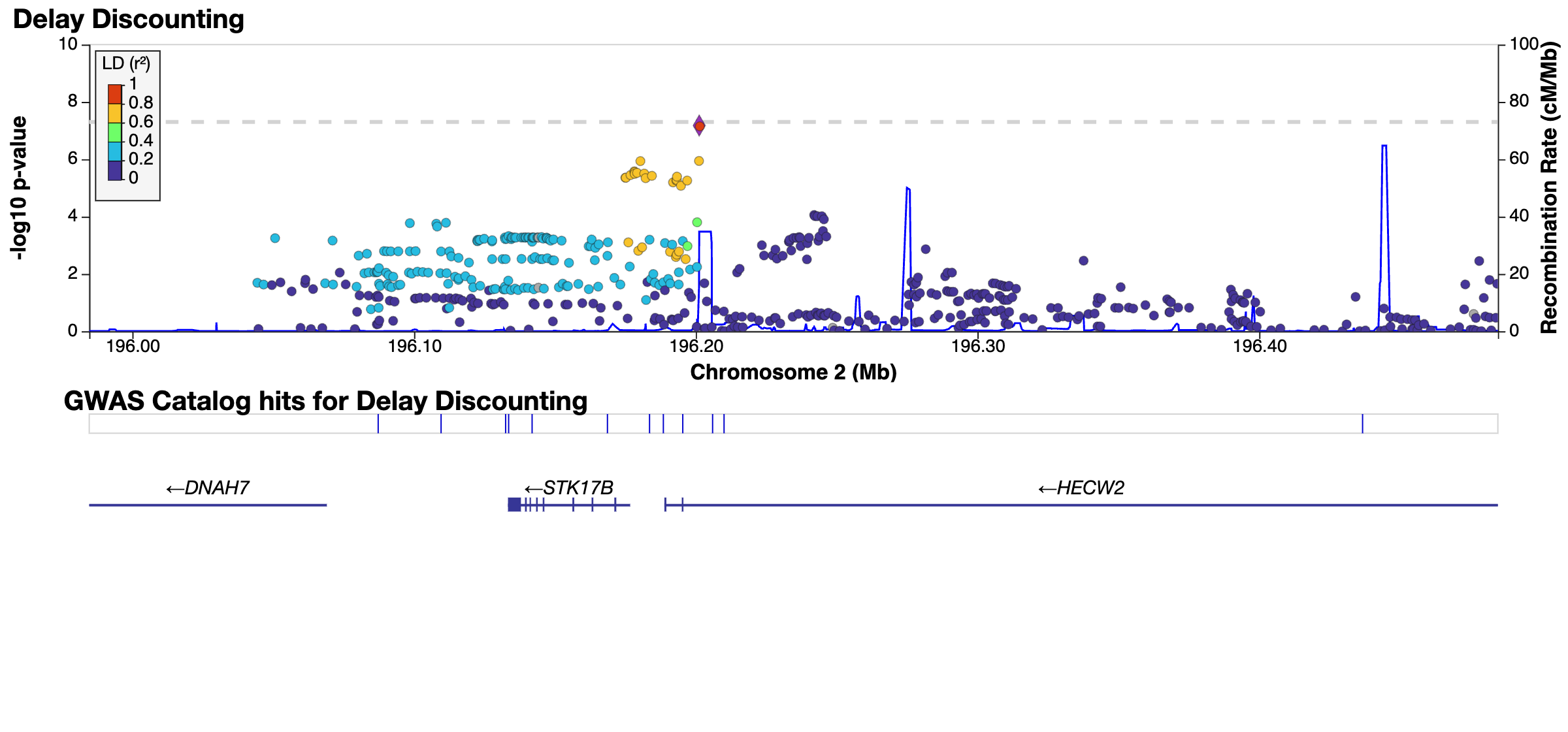

Supplement: Supplementary file 1 — Data S1: Supporting Information. [file GBB-24-e70033-s001.zip › GBB_70033_f8_SF8-locuszoom-HECW2.png]

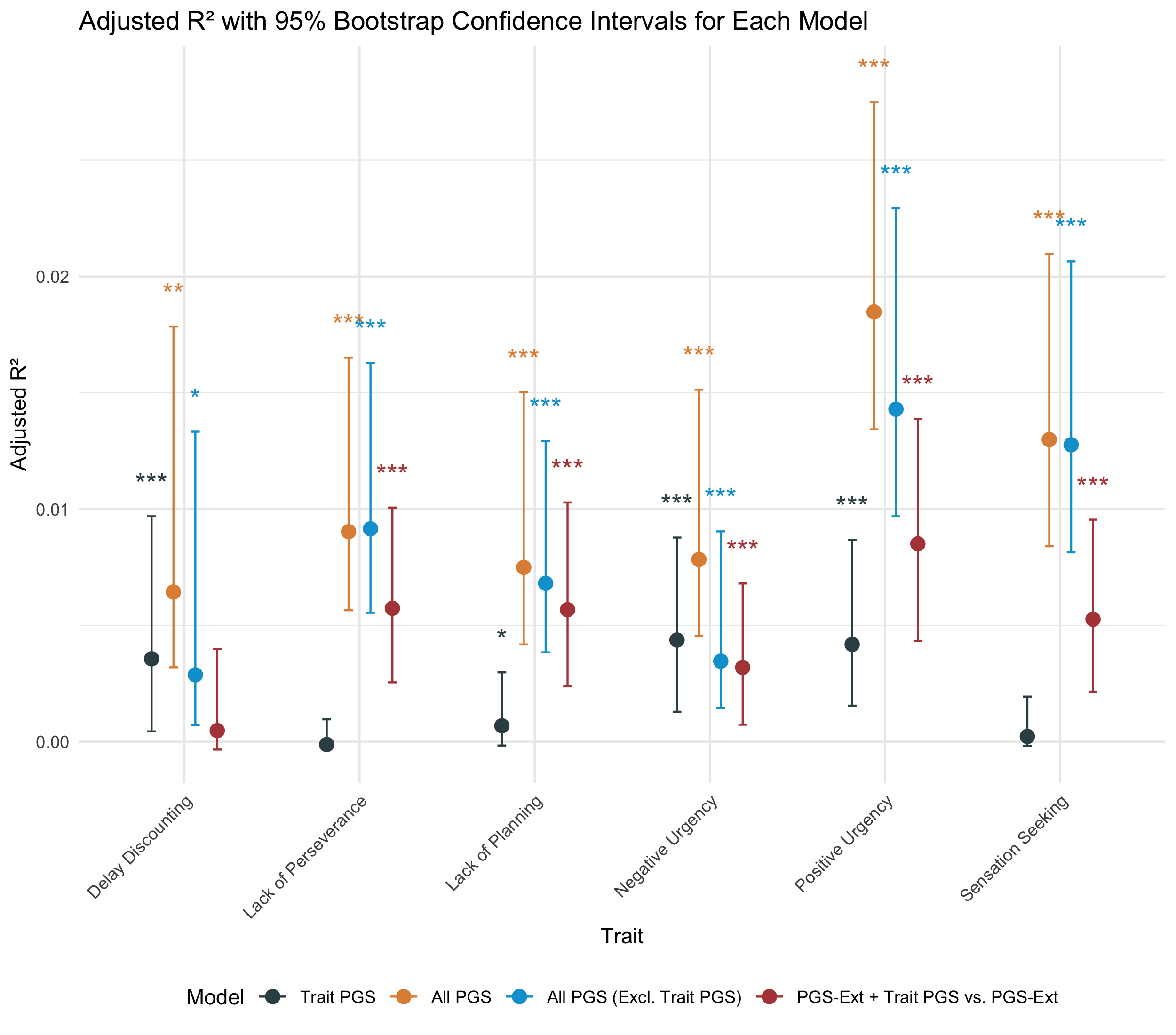

Supplement: Supplementary file 1 — Data S1: Supporting Information. [file GBB-24-e70033-s001.zip › GBB_70033_f9_SF9-Figure-comparePRS-March2025.png]

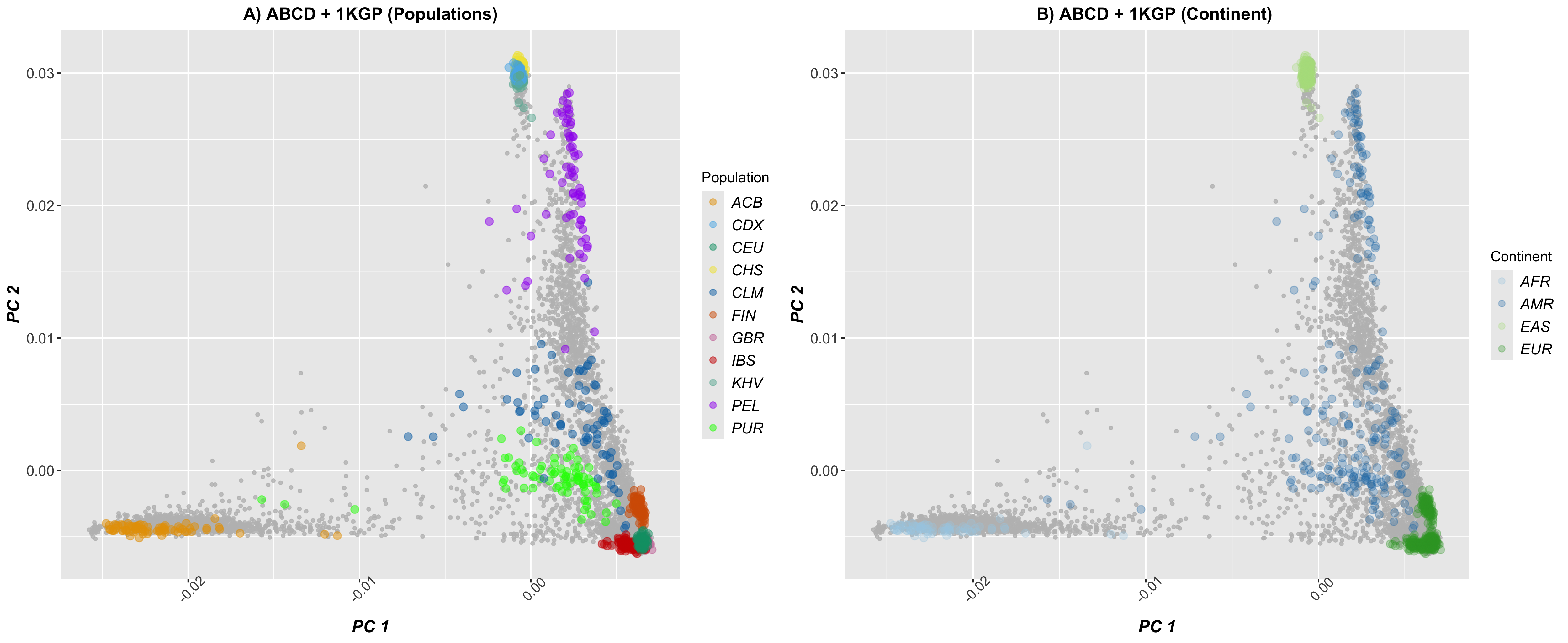

Supplement: Supplementary file 1 — Data S1: Supporting Information. [file GBB-24-e70033-s001.zip › gbb70033-sup-0001-FigureS1@FigureS1-R1.png]

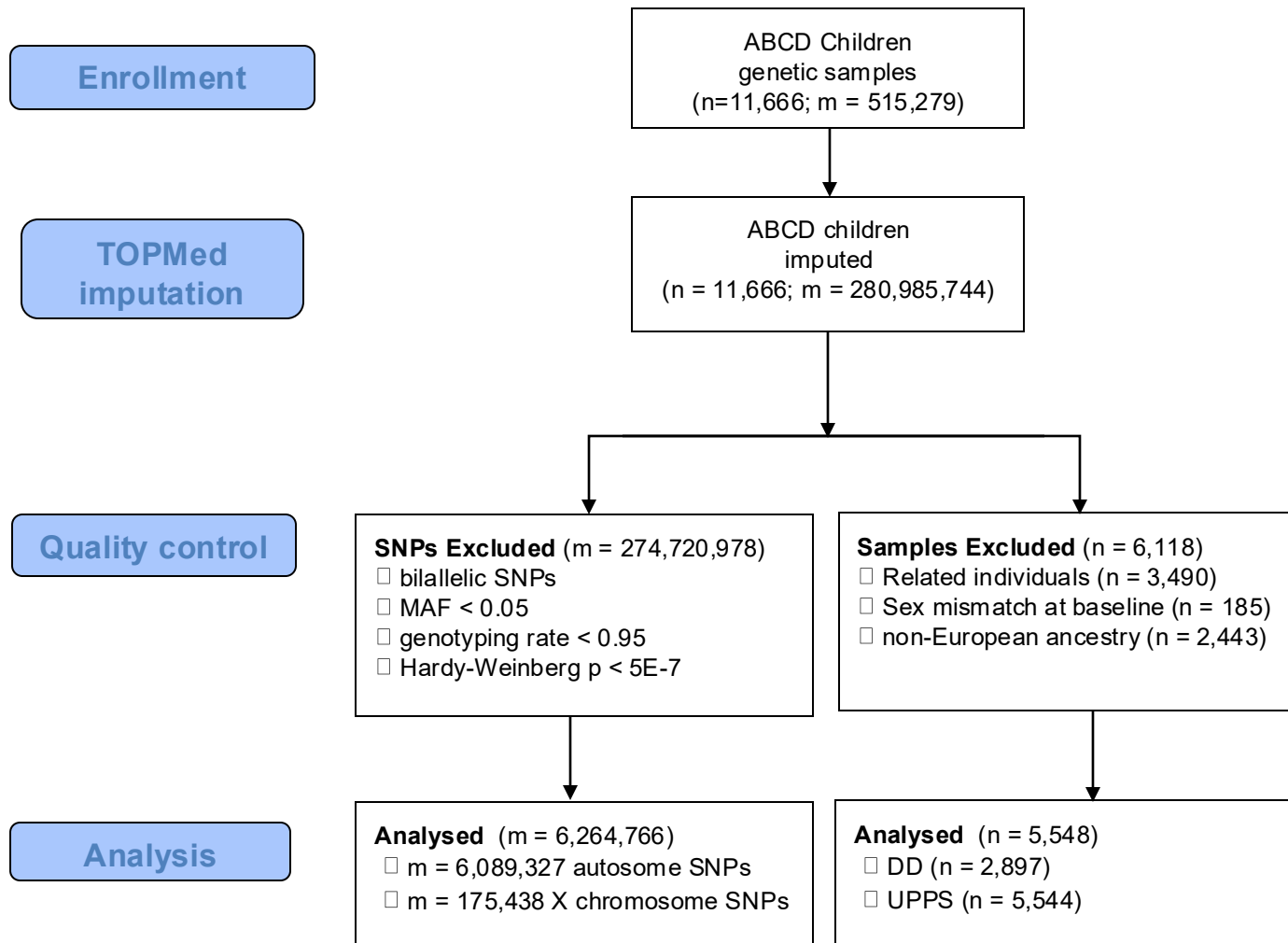

Supplement: Supplementary file 1 — Data S1: Supporting Information. [file GBB-24-e70033-s001.zip › gbb70033-sup-0003-FigureS2@FigureS2-R1.pdf]
